# Plasma levels of DPP4 activity and sDPP4 are dissociated from inflammation in mice and humans

**DOI:** 10.1038/s41467-020-17556-z

**Published:** 2020-07-28

**Authors:** Laurie L. Baggio, Elodie M. Varin, Jacqueline A. Koehler, Xiemin Cao, Yuliya Lokhnygina, Susanna R. Stevens, Rury R. Holman, Daniel J. Drucker

**Affiliations:** 1 Department of Medicine, Lunenfeld-Tanenbaum Research Institute, Mt. Sinai Hospital, Toronto, ON Canada; 2 Duke Clinical Research Institute, Duke University, Durham, NC USA; 3 Diabetes Trials Unit, Radcliffe Department of Medicine, University of Oxford, Oxford, UK

**Keywords:** Physiology, Endocrinology

## Abstract

Dipeptidyl peptidase-4 (DPP4) modulates inflammation by enzymatic cleavage of immunoregulatory peptides and through its soluble form (sDPP4) that directly engages immune cells. Here we examine whether reduction of DPP4 activity alters inflammation. Prolonged DPP4 inhibition increases plasma levels of sDPP4, and induces sDPP4 expression in lymphocyte-enriched organs in mice. Bone marrow transplantation experiments identify hematopoietic cells as the predominant source of plasma sDPP4 following catalytic DPP4 inhibition. Surprisingly, systemic DPP4 inhibition increases plasma levels of inflammatory markers in regular chow-fed but not in high fat-fed mice. Plasma levels of sDPP4 and biomarkers of inflammation are lower in metformin-treated subjects with type 2 diabetes (T2D) and cardiovascular disease, yet exhibit considerable inter-individual variation. Sitagliptin therapy for 12 months reduces DPP4 activity yet does not increase markers of inflammation or levels of sDPP4. Collectively our findings dissociate levels of DPP4 enzyme activity, sDPP4 and biomarkers of inflammation in mice and humans.

## Abstract (web summary)

DPP4 inhibitors are used for the treatment of diabetes, but the impact of DPP4 activity and soluble DPP4 on development of diabetes-associated inflammation remains uncertain. Here the authors study whether DPP4 inhibition controls sDPP4 and inflammatory biomarkers, and demonstrate that DPP4 inhibition is dissociated from changes in inflammation in mice and humans.

## Introduction

Dipeptidyl peptidase-4 (DPP4) is a ubiquitous serine protease that exists as either a membrane localised protein, or as a soluble form (sDPP4) that lacks transmembrane and cytosolic domains but retains catalytic activity and is found widely distributed in plasma and body fluids^1^. The aminopeptidase activity of DPP4 enables cleavage of dipeptides from the amino terminus of oligopeptides or small proteins, thereby modifying their activity. Notably, DPP4 cleaves and inactivates the incretin hormones, glucagon-like peptide-1 (GLP-1) and glucose-dependent insulinotropic polypeptide (GIP), terminating their ability to stimulate glucose-dependent insulin secretion^2,3^. Notwithstanding the putative importance of DPP4 for cleavage of multiple substrates with pleiotropic biological activities^4,5^, multiple DPP4 inhibitors are now widely used for the treatment of type 2 diabetes (T2D)^6^.

DPP4 was originally described as a lymphocyte cell surface protein, designated cluster of differentiation 26 (CD26). Indeed, DPP4 plays important roles in the maturation and activation of T-cells and immune responses, often acting independent of its catalytic activity^7–9^. Coincidentally, membrane-associated DPP4 is a receptor for Middle East Respiratory Syndrome coronavirus infection^10^. A complementary literature identifies sDPP4, shed from the cell surface, as a potential mediator of inflammation. Indeed, sDPP4 released from hepatocytes or adipose tissue, or administered exogenously, promotes inflammation in multiple tissues, often associated with the development of insulin resistance^9,11–17^.

The importance of inflammation for the development of β-cell failure and diabetes-associated complications^18^ has heightened interest in understanding whether glucose-lowering agents independently engage pathways governing inflammation^19^. While GLP-1 receptor (GLP-1R) agonists are thought to reduce inflammation^20^, whether DPP4 inhibitors modulate inflammation appears less clear. DPP4 inhibitors reduce pro-inflammatory cytokine production and immune cell infiltration in multiple tissues in both animals and humans^21–26^. On the other hand, several studies failed to detect an anti-inflammatory effect of DPP4 inhibitors in animals^27^ or humans^28,29^. Surprisingly, sustained inhibition of DPP4 activity in mice was associated with elevations in levels of circulating sDPP4, a molecule with pro-inflammatory activity^16^, without evidence for increased inflammation^17^, raising further questions about the relationships between DPP4 activity, sDPP4 and inflammation.

To better understand the relationship between DPP4 activity, sDPP4 and inflammation, we herein examine the consequences of DPP4 inhibition on DPP4 activity, levels of sDPP4 and biomarkers of inflammation in multiple tissues, including the plasma compartment of mice fed a high fat, fructose and cholesterol diet (HFFC) and in humans with T2D and established cardiovascular disease^30^. Furthermore, we use bone marrow transplant experiments to identify the source of elevated sDPP4 levels pursuant to pharmacological inhibition of DPP4 activity. In contrast to findings in mice, sustained inhibition of DPP4 activity with sitagliptin in humans is not associated with increases in circulating sDPP4 protein levels nor generalised changes in plasma biomarkers of inflammation. Moreover, sDPP4 levels vary widely from baseline to 12 months in these human subjects with T2D. Hence, continuous inhibition of DPP4 activity does not have a major impact on biomarkers of tissue and systemic inflammation in mice or in humans with established cardiovascular disease and T2D.

## Results

### DPP4 inhibition selectively increases DPP4 protein in tissue

**Fig. 1:**
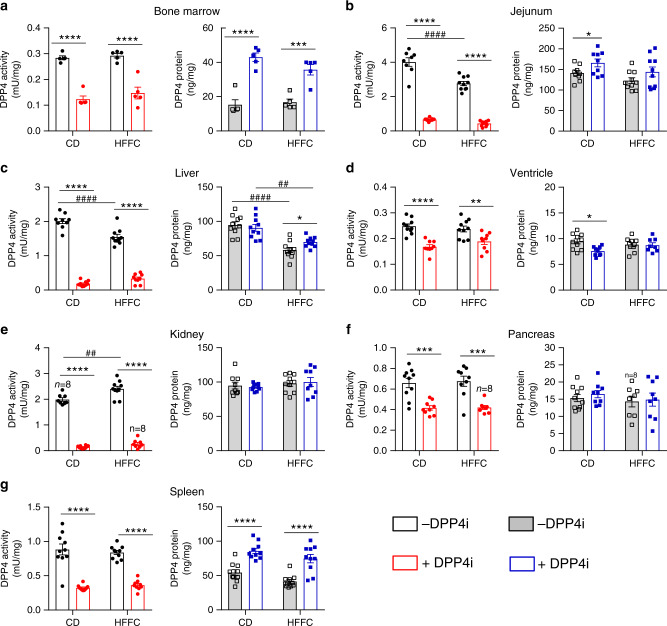
DPP4 activity and protein levels in tissues after 14 weeks of DPP4i. Levels of DPP4 activity (left panels) and protein (right panels) in **a** bone marrow, **b** jejunum, **c** liver, **d** whole ventricle, **e** kidney, **f** pancreas and **g** spleen from WT mice that were maintained on control diet (CD) or a high fat, fructose and cholesterol (HFFC) diet for 10 weeks and then the same diets supplemented with (+) or without (−) the DPP4 inhibitor (DPP4i) MK-0626 for 14 weeks. Data shown are mean ± SEM. For **a**, *n* = 5 mice per group. For **b**–**g**, *n* = 9 or 10 mice per group, except where *n* = 8 as indicated on the graph above the data set. *P < 0.05, **P < 0.01, ***P < 0.001, ****P < 0.0001 vs. DPP4i-treated. ^##^*P* < 0.01, ^####^*P* < 0.0001 for CD vs. HFFC. Data were analysed by two-way ANOVA. Source data are provided as a Source data file.

We recently demonstrated that the highly selective DPP4 inhibitors sitagliptin, linagliptin and MK-0626 rapidly increased plasma levels of sDPP4 protein in mice^17^. To ascertain the tissue sources contributing to the increase in circulating sDPP4, we measured levels of DPP4 activity and DPP4 protein in bone marrow, lung, jejunum, liver, cardiac ventricles, kidney, pancreas and spleen from WT mice fed a control diet (CD) or a high fat, fructose and cholesterol diet (HFFC) with or without the addition of the selective DPP4 inhibitor (DPP4i), MK-0626^27,31^ (Supplementary Fig. 1A). DPP4i treatment increased plasma levels of active GIP and GLP-1 in CD- and HFFC diet-fed mice (Supplementary Fig. 1B). Mice treated with the DPP4i for 14 weeks exhibited reductions in DPP4 activity in all tissues examined (Fig. 1) and increased levels of DPP4 protein in bone marrow, jejunum, liver and spleen (Fig. 1a–c, g). In contrast, DPP4 protein levels were unchanged, or slightly reduced in cardiac ventricles, kidney and pancreas (Fig. 1d–f). Analysis of earlier time points (Supplementary Fig. 2) demonstrated similar findings, with DPP4i-associated induction of DPP4 protein detected in organs, including lung, characterised by subpopulations of immune cells, principally lymphocytes.

### DPP4i-dependent increases in sDPP4 derive from bone marrow

**Fig. 2:**
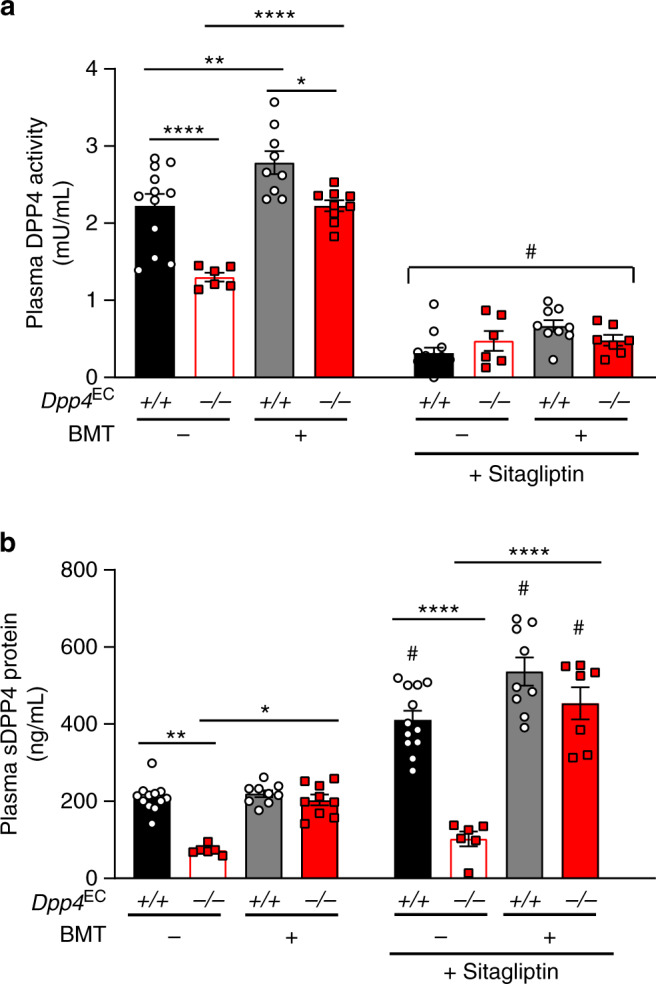
Bone-marrow-derived cells are the major source of DPP4 inhibitor-mediated increases in sDPP4. Plasma DPP4 activity **a** and sDPP4 protein levels **b** were measured 9 weeks following bone marrow transplant (BMT) or control procedure in control mice *(Dpp4*^EC+/+^) and mice with hematopoietic- and endothelial-specific inactivation of DPP4 (*Dpp4*^EC−/−^) after 7 days of free access to normal drinking water or water containing sitagliptin. Data shown are mean ± SEM. *n* = 12 *Dpp4*^EC+/+^– BMT, 9 *Dpp4*^EC+/+^+ BMT, 6 *Dpp4*^EC−/−^ – BMT and 7 or 9 for *Dpp4*^EC−/−^ + BMT with or without sitagliptin, respectively. *P < 0.05, **P < 0.01, ****P < 0.0001 vs. the indicated comparator. ^#^*P* < 0.01–0.0001 vs. the respective non-sitagliptin-treated group. All data were analysed by one-way ANOVA. Source data are provided as a Source data file.

Studies in mice demonstrated that DPP4 expression within Tie2+ hematopoietic or endothelial cells contributes to the increased levels of circulating sDPP4 observed following enzymatic inhibition of DPP4 activity^17^. To differentiate between endothelial or hematopoietic sources of sDPP4, control mice (*Dpp4*^EC+/+^) and mice with loss of *Dpp4* expression specifically within Tie2+ cells (representing cells of endothelial or hematopoietic lineage; *Dpp4*^EC−/−^) received a bone marrow transplant (BMT) from WT mice or underwent a sham BMT procedure. Eight weeks following BMT, mice were given free access to drinking water with or without the DPP4i sitagliptin for 7 days, after which plasma DPP4 activity and sDPP4 protein levels were measured. Basal levels of plasma DPP4 activity and sDPP4 were reduced in *Dpp4*^EC−/−^ mice compared to *Dpp4*^EC+/+^ controls (Fig. 2a, b), consistent with important contribution of the Tie2+ compartment towards circulating DPP4^32^. However, levels of both DPP4 activity and sDPP4 protein were normalised in *Dpp4*^EC−/−^ mice that received WT bone marrow (Fig. 2a, b). Sitagliptin treatment reduced plasma DPP4 activity and increased levels of sDPP4 protein regardless of BMT status in *Dpp4*^EC+/+^ control mice (Fig. 2a, b). In contrast, although sitagliptin reduced plasma DPP4 activity, sDPP4 levels were not increased in *Dpp4*^EC−/−^ mice that did not undergo BMT (Fig. 2a, b). However, BMT restored the actions of sitagliptin to increase circulating sDPP4 in *Dpp4*^EC−/−^ mice (Fig. 2b). Collectively, these data illustrate that the bone marrow contributes substantially to basal levels of sDPP4 and that increased levels of sDPP4 protein detected in mouse plasma following DPP4i treatment originate predominantly from bone-marrow-derived Tie2+ hematopoietic cells.

### DPP4 inhibition does not modify inflammation in mice

**Fig. 3:**
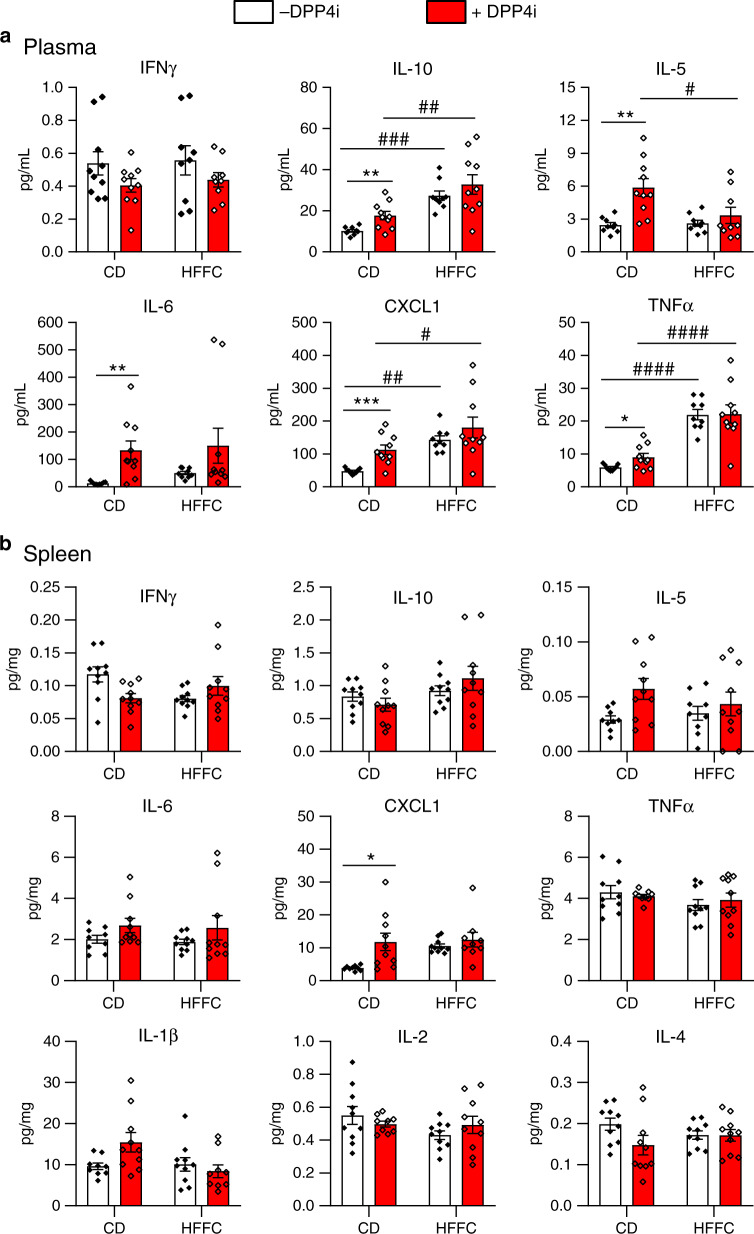
Cytokine levels in mouse plasma and spleen after 14 weeks of the DPP4 inhibitor MK-0626. Levels of inflammatory markers in **a** plasma and **b** spleen of WT mice that were maintained on control diet (CD) or a high fat, fructose and cholesterol (HFFC) diet for 10 weeks and then the same diets supplemented with (+) or without (−) the DPP4 inhibitor (DPP4i) MK-0626 for 14 weeks. Data shown are mean ± SEM. *n* = 9 or 10 mice per group. *P < 0.05, **P < 0.01, ***P < 0.001 vs. DPP4i-treated. ^#^*P* < 0.05, ^##^*P* < 0.01, ^###^*P* < 0.001, ^####^*P* < 0.0001 for CD vs. HFFC. Data were analysed by two-way ANOVA. Source data are provided as a Source data file.

**Fig. 4:**
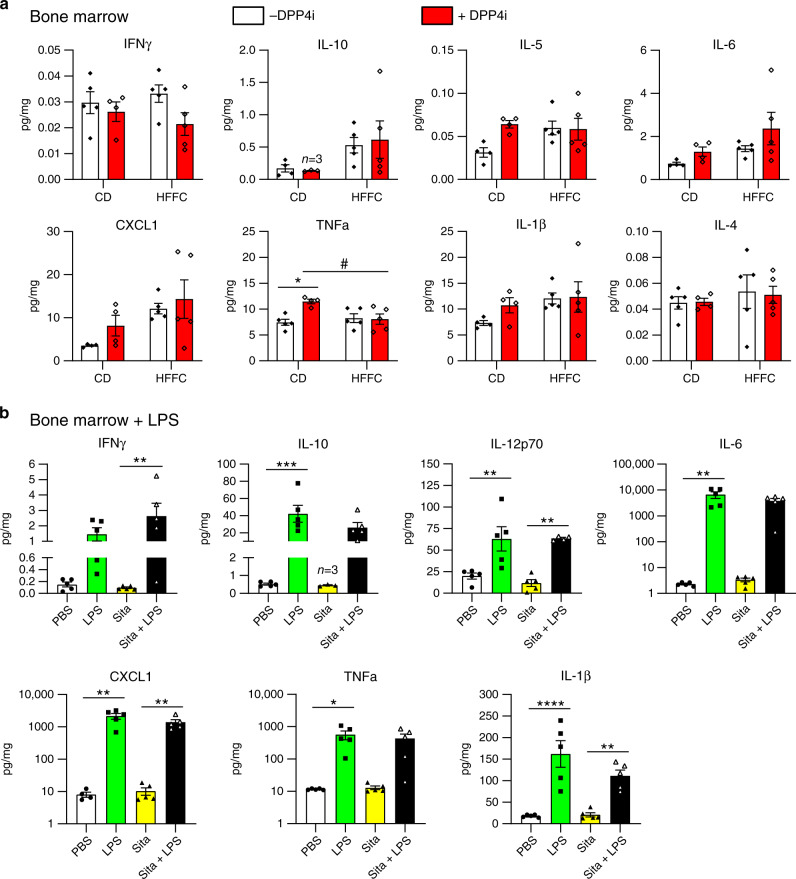
Cytokine levels in mouse bone marrow after DPP4i treatment and LPS challenge. Levels of inflammatory markers in bone marrow from WT mice that were **a** maintained on control diet (CD) or a high fat, fructose and cholesterol (HFFC) diet for 10 weeks and then the same diets supplemented with (+) or without (−) the DPP4 inhibitor (DPP4i) MK-0626 for 14 weeks or **b** fed a HFFC diet for 4 days and treated with LPS (two injections of 35 μg each, one on the evening prior to sacrifice and the second 1 h before sacrifice) or PBS vehicle. Data shown are mean ± SEM. *n* = 4 or 5 mice per group, except where *n* = 3 as indicated on the graph above the data set. *P < 0.05, **P < 0.01, ***P < 0.001, ****P < 0.0001 vs. DPP4i- or LPS-treated. ^#^*P* < 0.05 for CD vs. HFFC. Data were analysed by two-way ANOVA. Source data are provided as a Source data file.

To probe for a putative link between DPP4 activity, sDPP4 and inflammation, we assessed plasma levels of inflammatory cytokines in mice fed a CD or HFFC diet for 10 weeks, with or without subsequent addition of MK-0626 for an additional 14 weeks (Supplementary Fig. 1A). Surprisingly, DPP4 inhibition increased plasma levels of IL-10, IL-5, IL-6, CXCL1 and TNFα in CD-fed mice, whereas levels of IL-10, CXCL1 and TNFα were increased with HFFC diet feeding, but not further increased by MK-0626 (Fig. 3a). To determine whether similar pro-inflammatory changes were detected in key tissues demonstrating local DPP4i-associated upregulation of DPP4 protein, we analysed cytokine levels in spleen (Fig. 3b) and bone marrow (Fig. 4). Only CXCL1 was increased in splenic extracts from CD-fed mice treated with MK-0626, whereas levels of IFNγ, IL-10, IL-5, IL-6, TNFα, IL-1β, IL-2 and IL-4 were not different in CD vs. HFFC-fed mice (Fig. 3b), despite substantial reduction of splenic DPP4 activity (Fig.1g). Similarly, only TNFα was increased in the bone marrow of CD-fed mice treated with MK-0626, whereas IFNγ, IL-10, IL-5, IL-6, CXCL1, IL-1β, and IL-4 were not different in CD vs. HFFC-fed mice (Fig. 4a), despite simultaneous reduction of DPP4 activity and induction of DPP4 protein levels (Fig. 1a).

We next examined whether exposure to an inflammatory mediator such as bacterial-derived lipopolysaccharide (LPS) could unmask effects of DPP4 inhibitor treatment on inflammation. Accordingly, we measured levels of inflammatory markers in bone marrow from WT mice that were fed a HFFC diet with or without the DPP4i sitagliptin for 3 days and then challenged with LPS or vehicle. Sitagliptin reduced DPP4 activity and increased DPP4 protein levels in both plasma and bone marrow from control and LPS-treated mice (Supplementary Fig. 3). However, although LPS administration robustly increased cytokine levels in bone marrow, sitagliptin did not modify the induction of bone marrow cytokine levels in LPS-treated mice (Fig. 4b).

To further assess whether modulation of DPP4 activity and sDPP4 were associated with changes in markers of tissue inflammation, we measured mRNA levels of genes linked to inflammation in multiple tissues. Levels of these mRNA transcripts were generally higher in liver, but not other tissues, in HFFC vs. CD-fed mice (Supplementary Figs. 4–7). Inhibition of DPP4 activity for 2 weeks was associated with increased levels of some inflammatory markers in liver and kidney (Supplementary Fig. 6), whereas transcript levels of these inflammatory genes were not different or reduced in these tissues following sustained inhibition of DPP4 activity for 14 weeks (Supplementary Fig. 4). Nevertheless, exposure to MK-0626 for 2 or 14 weeks did not consistently alter expression of genes associated with inflammation in liver, kidney, jejunum or spleen (Supplementary Figs. 4–7).

### *Glp1r* loss does not alter the impact of DPP4i on inflammation

We previously demonstrated that GLP-1R agonism reduces gut inflammation, whereas *Glp1r*^−/−^ mice were more susceptible to intestinal injury and exhibited dysregulation of intestinal mRNA transcripts corresponding to genes important for immune regulation, epithelial protection and repair^33,34^. Moreover, DPP4 inhibitors augment levels of active GLP-1 and also reduce gut inflammation^35,36^. As GLP-1, acting through the GLP-1 receptor, is the most extensively validated target for DPP4, and a DPP4 substrate consistently linked to the reduction of inflammation^1,37^, we postulated that anti-inflammatory actions arising from DPP4 inhibition would be mediated by GLP-1. Hence, we examined the putative anti-inflammatory actions of MK-0626 in *Glp1r*^−/−^ mice fed a HFFC diet (Supplementary Fig. 8A). HFFC feeding increased body weight and MK-0626 treatment decreased DPP4 activity and increased DPP4 protein levels in plasma and bone marrow samples from both *Glp1r*^*+/+*^ and *Glp1r*^−/−^ mice (Supplementary Fig. 8B–D). Levels of *Il10*, *Il25*, *Reg3g* and *Tlr4* mRNA transcripts were lower in the ileum and *Tff2* mRNA transcripts were reduced in the colon of *Glp1r*^−/−^ mice (Supplementary Fig. 9). Nevertheless, no consistent inflammatory gene expression signatures were detected in the jejunum, ileum or colon of *Glp1r*^*+/+*^ and *Glp1r*^−/−^ mice following systemic inhibition of DPP4 activity (Supplementary Fig. 9). Hence, basal activity of the GLP-1R^34^, but not reduction of DPP4 activity, modulates expression of a subset of inflammation-related genes in the mouse intestine.

### Sitagliptin does not alter sDPP4 or cytokines in T2D humans

**Fig. 5:**
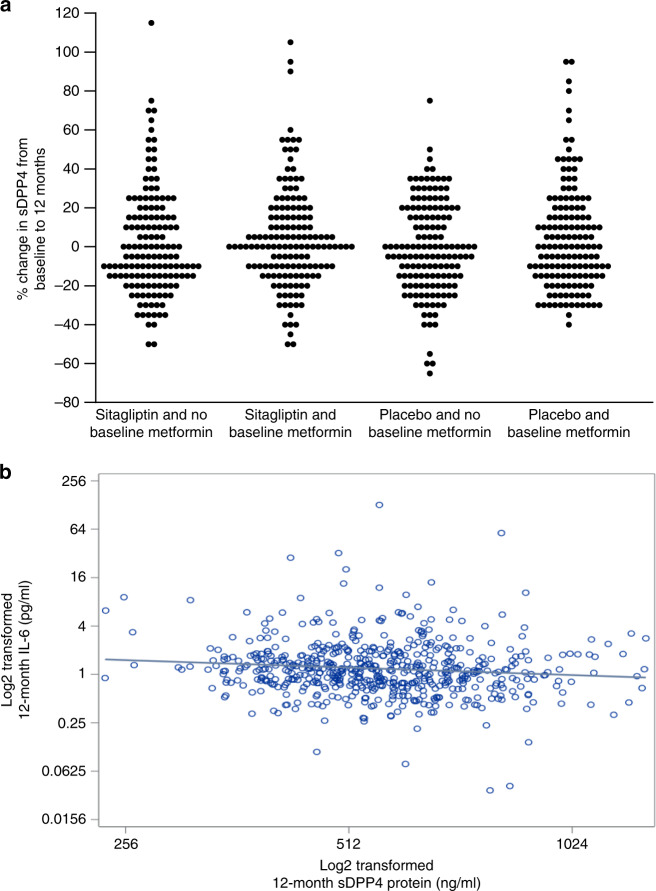
Sitagliptin does not increase plasma sDPP4 levels in humans with T2D. **a** Percent change in individual sDPP4 levels from baseline to 12-months in TECOS trial plasma samples from T2D patients that were treated with or without sitagliptin. Percent change in sDPP4 levels was categorised to the nearest 5% and the frequency of each category plotted separately. **b** Correlation between plasma levels of 12-month IL-6 and sDPP4. All data are log2 transformed.

In contrast to extensive studies of DPP4 and inflammation in animals, less is known about whether prolonged exposure to DPP4 inhibitors modifies circulating markers of inflammation or sDPP4 in humans with T2D. Hence, we analysed DPP4 activity, sDPP4 protein and levels of inflammatory markers in plasma samples from 600 subjects with T2D treated with or without sitagliptin for 12 months from the TECOS trial, a study examining the safety of sitagliptin in subjects with T2D and established cardiovascular disease^30^. Baseline characteristics, including HbA_1c_, BMI, plasma levels of DPP4 activity and sDPP4, as well as plasma markers of inflammation were not clinically different in those randomised to sitagliptin vs. placebo (Supplementary Table 1). Interestingly, individuals receiving metformin had lower baseline levels of sDPP4, DPP4 activity, CRP, IL-6, TNF-α and MCP-1 (Supplementary Table 2). Plasma DPP4 activity was markedly reduced at 12 months in sitagliptin-treated subjects (Table 1). Nevertheless, sDPP4 protein levels were not different at 12 months in subjects receiving sitagliptin (Table 1), with individual patient level data for change in sDPP4 shown in Fig. 5a. Moreover, we detected no differences, relative to baseline values, in circulating levels of IL-6, TNFα and CRP at 12 months, however MCP-1 levels were lower in people treated with sitagliptin (Table 1). Furthermore, an examination of correlations between levels of sDPP4 and plasma cytokines at 12 months detected modestly lower levels of IL-6 with increasing levels of sDPP4 (Fig. 5b and Supplementary Table 3). Nevertheless, no other correlations were detected between sDPP4 and circulating markers of inflammation after 12 months of therapy (Supplementary Table 3).

**Table 1:** Association of sitagliptin and 12-month biomarkers adjusted for baseline biomarker and other covariates.

12-month log base-2 transformed biomarker	*N*	Least squares means for placebo	Least squares means for sitagliptin	Difference in least squares means	*F*	*p* value
sDPP4	592	9.15 (9.11, 9.18)	9.13 (9.09, 9.17)	−0.02 (−0.07, 0.04)	0.4	0.528
DPP4 activity	591	1.83 (1.71, 1.95)	−0.52 (−0.64, −0.40)	−2.35 (−2.51, −2.18)	775.32	**<0.001**
CRP	592	1.33 (1.18, 1.49)	1.23 (1.07, 1.39)	−0.11 (−0.33, 0.12)	0.85	0.357
IL-6	591	0.23 (0.13, 0.33)	0.24 (0.14, 0.34)	0.01 (−0.13, 0.15)	0.01	0.918
TNFa	592	1.47 (1.43, 1.52)	1.48 (1.43, 1.52)	0.00 (−0.06, 0.07)	0.01	0.920
MCP-1	592	7.49 (7.45, 7.53)	7.42 (7.38, 7.46)	−0.07 (−0.13, −0.01)	4.85	**0.028**

Statistically significant *p* values are indicated by bold print. *P* values are two-sided and derived from multiple linear regression models. No adjustments were made for multiple comparisons.

### Metformin attenuates sitagliptin-induced increases in DPP4

**Fig. 6:**
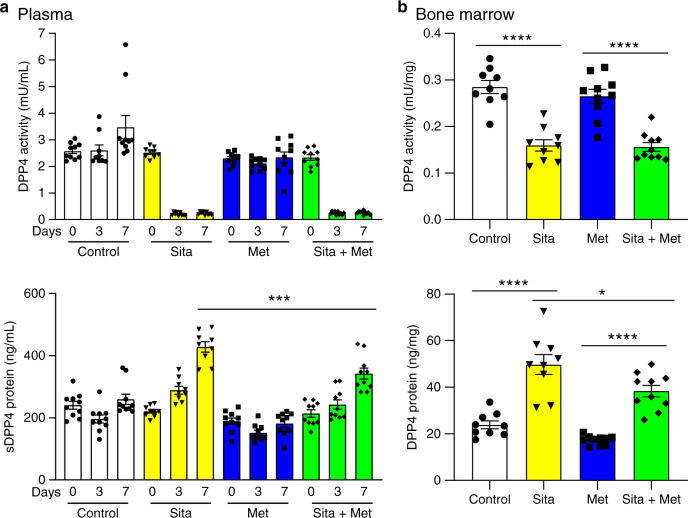
Metformin reduces DPP4 protein levels in sitagliptin-treated mice. **a** DPP4 activity (upper panel) and sDPP4 protein concentration (lower panel) in mouse plasma before (0) and after 3 and 7 days of free access to normal drinking water and HFFC diet (Control), normal drinking water and HFFC diet containing sitagliptin (Sita), drinking water supplemented with metformin and HFFC diet (Met), or drinking water supplemented with metformin and HFFC diet containing sitagliptin (Sita + Met). **b** DPP4 activity (upper panel) and DPP4 protein levels (lower panel) in mouse bone marrow after 7 days of free access to normal drinking water and HFFC diet (Control), normal drinking water and HFFC diet containing sitagliptin (Sita), drinking water supplemented with metformin and HFFC diet (Met), or drinking water supplemented with metformin and HFFC diet containing sitagliptin (Sita + Met). Data shown are mean ± SEM. *n* = 9 or 10 mice per group. *P < 0.05, ***P < 0.001, ****P < 0.0001 vs. Sita or Sita + Met, as indicated. Data were analysed by one-way ANOVA. Source data are provided as a Source data file.

Circulating levels of sDPP4 and DPP4 activity have been reported as elevated in people with T2D, correlated with BMI and reduced in subjects treated with metformin^38,39^. As metformin does not directly inhibit DPP4 enzymatic activity^40,41^, these findings have been attributed to a metformin-mediated reduction in total sDPP4 levels^38,40,42^. To probe how metformin regulates sDPP4, we measured DPP4 activity and sDPP4 protein levels in mice that were treated with a combination of sitagliptin and metformin and compared these parameters in mice treated with either agent alone. Combined sitagliptin + metformin administration reduced levels of plasma sDPP4 protein relative to those detected with sitagliptin alone (Fig. 6a). Furthermore, the combination of sitagliptin + metformin was associated with a decreased induction of DPP4 protein levels in bone marrow, when compared to sitagliptin treatment alone (Fig. 6b). These findings further localise the dynamic regulation of sDPP4 by DPP4 inhibitors to the bone marrow compartment and are consistent with observations that sDPP4 levels were lower at baseline in metformin-treated human subjects studied in the TECOS trial (Supplementary Table 2).

## Discussion

DPP4 is a widely expressed enzyme; hence potential sources of circulating sDPP4 include endothelial cells, liver, kidney, adipose tissue, enterocytes, smooth muscle cells and hematopoietic cells, including lymphocytes. Circulating levels of DPP4 activity and sDPP4 are increased in humans with T2D, possibly secondary to increased shedding of sDPP4 from adipocytes^43^, and peripheral blood mononuclear cells, predominantly T cells^44^. Studies of individuals with congenital lymphocyte deficiency have implicated lymphocytes as an important cellular source for circulating levels of sDPP4 in humans^45^, consistent with our detection of reduced circulating sDPP4 in mice with *Tie2*-Cre-mediated inactivation of *Dpp4*^17^. Here we identify the bone marrow compartment as a key source of increased sDPP4 levels following administration of DPP4 inhibitors in mice, a cell population also targeted by metformin which reduces sDPP4 levels in mice and humans. Nevertheless, the mechanisms linking reduced DPP4 activity to increased hematopoietic cell-derived sDPP4, and the biological impact of these changes in the bone marrow and beyond, requires further investigation.

Our current findings using BMT implicate the bone marrow, and not adipocytes or endothelial cells, as a critical source for the increase in circulating sDPP4 observed following inhibition of the DPP4 enzyme. These observations are consistent with results of BMT studies implicating hematopoietic cells as critical for restoring basal levels of circulating sDPP4 in *Dpp4*^−/−^ mice^45^. Similar conclusions support the importance of bone-marrow-derived hematopoietic cells as the dominant source of circulating sDPP4 following either kidney transplantation or BMT in DPP4-deficient rats^46^. The mechanisms underlying the unique susceptibility of bone-marrow-derived cells, including lymphocytes, to enhanced release of sDPP4 following catalytic DPP4 inhibition are not known and deserve further investigation.

The rationale for development of DPP4 inhibitors for the therapy of T2D was largely based on findings that DPP4 inactivated GLP-1 and GIP, whereas inhibition of the enzyme stabilised levels of active incretin hormones and enhanced glucose homeostasis^1,3,47,48^. As inflammation is thought to underlie many of the complications associated with T2D, multiple studies have examined whether changes in inflammation arise following use of DPP4 enzyme inhibitors in the treatment of T2D^16,21,28,49–52^. Nevertheless, an emerging complementary literature, based predominantly on studies in cells and mice, has focused on the putative pro-inflammatory role of sDPP4 as an adipokine or hepatokine, independent of its catalytic activity^9,11,12,16^.

Interestingly, the combination of sitagliptin + metformin was associated with a reduced induction of bone marrow DPP4 protein levels vs. sitagliptin treatment alone. These data support results from our bone marrow transplant experiments, identifying the bone marrow compartment as the source of increased levels of plasma sDPP4 detected following DPP4 inhibitor treatment. Moreover, our observations demonstrate that metformin treatment consistently reduces levels of sDPP4 protein in both mice and humans. Whether metformin predominantly reduces circulating sDPP4 by modulating DPP4 synthesis or shedding in specific tissue pools such as the bone marrow, or by also enhancing sDPP4 clearance, remains uncertain.

Although sDPP4 directly induces inflammation in cells and animals^11,15,53^, whether circulating sDPP4 levels reflect the extent of localised or tissue inflammation is unclear. Levels of sDPP4 were reduced in humans with acute HIV infection and did not change with prolonged antiretroviral therapy^54^. In contrast, multiple studies demonstrated elevated levels of DPP4 activity and sDPP4 in subjects with obesity and/or T2D^11,15,39^, associated in some analyses with increased sDPP4 expression in circulating T cells^39^. Interestingly, 24 weeks of dapagliflozin reduced levels of sDPP4 and liver enzymes in subjects with T2D, however levels of sDPP4 also decreased in the control group independent of changes in liver enzymes^55^.

The current studies examined whether inhibition of DPP4 activity, studied in animals with prolonged exposure to regular chow or high fat diets, or in humans with T2D and established cardiovascular disease, would be associated with altered inflammatory tone, as assessed through analysis of cytokine expression. Multiple reports have described reduced markers of tissue or systemic inflammation in animals treated with DPP4 inhibitors^1^. In contrast, we observed increased plasma levels of IL-10, IL-5, IL-6, CXCL1 and TNFα in CD-fed mice treated with MK-0626, however these differences were no longer apparent in HFFC-fed mice. We interpret these findings as consistent with sDPP4-mediated upregulation of tissue inflammation under conditions of low fat-diet-associated inflammatory tone, whereas the importance of sDPP4 for inflammation may no longer be apparent in the context of upregulated tissue inflammation in high-fat diet-fed animals. Moreover, we did not detect consistent alterations in cytokine expression within liver, kidney, intestine, spleen, or bone marrow following sustained inhibition of systemic and tissue DPP4 activity, despite increased levels of DPP4 protein. These findings are consistent with data demonstrating no effect of sitagliptin administration on visceral adipose tissue inflammation in mice^16^ and our recent data dissociating levels of DPP4 activity and sDPP4 from cytokine levels in plasma, liver, or adipose tissue of high-fat diet-fed mice treated with MK-0626^17^. Hence, the available preclinical data do not support a reproducible anti-inflammatory effect of DPP4 inhibitors in murine models of diet-induced inflammation.

Whether DPP4 inhibitors reduce inflammation in humans appears highly context- and study-dependent. A 3-month regimen of sitagliptin 50 mg once daily reduced circulating levels of SAA-LDL, C-reactive protein (CRP), and TNF-α in 24 subjects with T2D^21^, and sitagliptin therapy, 100 mg daily for 12 months, resulted in lower levels of resistin, vaspin, omentin-1 and TNF-α in subjects with T2D^56^. Similarly, sitagliptin reduced plasma levels of CRP, IL-6, IL-18, secreted phospholipase-A_2,_ soluble ICAM-1 and E-selectin in 36 subjects with T2D treated for 6 weeks^51^. Notably, many of these studies did not contain active comparator glucose-lowering controls and subjects treated with DPP4 inhibitors generally exhibited greater reductions in HbA_1c_ relative to placebo-treated controls. In contrast, administration of sitagliptin once daily for 24 weeks had no effect on numbers of CD4 + T cells, or plasma levels of RANTES and soluble TNF receptor II, despite reduction in glucose levels in HIV + subjects^57^. Similarly, 16 weeks of once daily sitagliptin therapy had no consistent effect on circulating markers of inflammation in non-diabetic subjects with HIV on stable antiretroviral therapy^58^.

Our current studies of inflammatory biomarkers from a subset of subjects in the TECOS trial over 12 months represent the largest cohort yet analysed for correlations between DPP4 activity, sDPP4 and biomarkers of inflammation. Consistent with the pharmacodynamic profile of sitagliptin, plasma levels of DPP4 activity were markedly reduced at 12 months in subjects randomised to sitagliptin. In contrast to observations in mice, circulating levels of sDPP4 were not increased in sitagliptin-treated subjects. Moreover, plasma sDPP4 levels varied widely from baseline to 12 months, and were lower at baseline in subjects treated with metformin. Furthermore, we did not observe changes in the majority of plasma cytokines in sitagliptin-treated subjects, and with the exception of IL-6, no direct correlation between sDPP4 and plasma cytokines was observed. Hence, these findings in mice and humans further dissociate changes in DPP4 activity from circulating levels of sDPP4, and plasma biomarkers of inflammation.

Intriguingly, a previous study examined changes in sDPP4 following administration of sitagliptin 100 mg once daily to 12 obese subjects (mean BMI 35.1) with T2D. Fasting plasma levels of sDPP4 increased rapidly when measured at 2 and 4 h following a single dose of sitagliptin, and were elevated when measured in the fasting state at 2, 4, 8 and 12 weeks of sitagliptin administration^23^. Nevertheless, despite simultaneous increases in circulating sDPP4, levels of mRNA transcripts for *TNFa*, *JNK1*, *IKKb*, *TLR4*, *TLR2* and *CCR2* were reduced in RNA isolated from circulating mononuclear cells at the same time points. Whether the increase in sDPP4 is attenuated over time in human studies with DPP4 inhibitors will require additional time course analyses.

Our studies have a number of limitations. First, we limited our analyses of inflammation to interrogation of a limited set of cytokines and RNA transcripts in mice with diet-induced inflammation, and we did not examine changes in the proportions of circulating or tissue-associated immune cell populations. Second, we did not study the relationships between DPP4 inhibition, sDPP4 and inflammation, in people with severe obesity, or those with pre-existing inflammatory conditions such as people with acute infections. Third, our analyses of human plasma samples from the TECOS trial were limited to two time points, baseline values and samples obtained at 12 months, and it is possible that putative relationships between DPP4 activity, sDPP4 and inflammatory biomarkers may be apparent earlier, after different durations of sitagliptin exposure. Moreover, the majority of assays utilised do not reliably discriminate total intact peptide substrate from DPP4-cleaved cytokines, precluding definitive assessments of relative changes in the bioactivity of the cytokines analysed in the context of treatment with DPP4 inhibitors.

To rule out the potential impact of different sDPP4 assays on interpretation of potential changes in plasma sDPP4, we assessed plasma sDPP4 levels in a subset of the same individuals using two additional sDPP4 ELISAs (Supplementary Fig. 10). Although absolute sDPP4 levels varied between assays, none of the assays revealed an induction of circulating sDPP4 levels following sitagliptin administration. Collectively, our findings provide extensive new information highlighting the unexpected inter-individual variability in baseline sDPP4 levels as well as fluctuation of circulating levels of sDPP4 over 12 months in people with T2D. Importantly, we provide new evidence that humans with T2D subjected to 12 months of sustained inhibition of DPP4 activity do not exhibit correlations between reduction of DPP4 activity, levels of sDPP4 and changes in biomarkers of inflammation. Hence, it seems unlikely that the failure to reduce the rates of cardiovascular outcomes in clinical trials examining the safety of DPP4 inhibitors is attributable to unanticipated links between DPP4 activity, sDPP4 and inflammation.

Since we initiated these studies, the importance of T2D as a risk factor for SARS-CoV-2 infection has emerged, generating additional questions surrounding the potential impact and safety of specific diabetes therapies in the context of acute coronavirus-associated inflammation^59^. The current findings extend our understanding of serial changes in inflammatory biomarkers in subjects with T2D treated with sitagliptin, and provide an important reference point for future analyses interrogating alterations in inflammation potentially linked to diabetes therapies in individuals with SARS-CoV-2-associated infection.

## Methods

### Mice

The animal studies described herein complied with all relevant ethical regulations for animal testing and research. The animal protocols were approved by the Animal Care Committee of Mt. Sinai Hospital through the Toronto Centre for Phenogenomics, Protocol Approval number 20-0045H. WT C57BL/6J male mice were purchased from The Jackson Laboratory (stock #000664) and acclimated to the animal facility for a minimum of one week. Male whole-body *Glp1r*^−/−^ mice on the C57BL/6 genetic background^60^ and *Glp1r*^*+/+*^ controls were generated by crossing *Glp1r*^*+/−*^ mice from the same litter or family. Female *Dpp4*^EC+/+^ and *Dpp4*^EC−/−^ mice were generated as described^17^. The data in Supplementary Fig. 2A are from control *Dpp4*^Hep+/+^ male mice (described in Varin et al.^17^) that were fed a high fat, fructose and cholesterol (HFFC) diet (described below) for 14 weeks or a HFFC diet for 10 weeks and then switched to HFFC diet containing the DPP4 inhibitor MK-0626 (described below) for 4 weeks. All mice were housed at a maximum of five mice per cage under specific pathogen-free conditions in microisolator cages and maintained on a 12-h light/dark cycle (07:00 a.m./07:00 p.m.) in a temperature (21 °C) and humidity-controlled environment with free access to rodent chow and water. Body weights were measured once a week in all mice.

### Mouse diets

All mice were initially maintained on regular chow (18% kcal fat, Harlan Teklad). At 6 weeks of age, WT C57BL/6J males were randomised to receive either a high fat (60% kcal fat, Research Diets #D12492) or control (10% kcal fat matched diet, Research Diets #D12450J) diet for 8 weeks. To induce inflammation, mice that were fed the 60% high-fat diet were switched to a high fat, fructose and cholesterol diet (HFFC; 40% kcal fat, 20% kcal fructose and 2% cholesterol, Research Diets #D09100301) for 10 weeks; whereas those mice that were fed the control diet were switched to the appropriate matching control diet (CD; 10% kcal fat, 0% fructose and 0% cholesterol, Research Diets #D09100304) for 10 weeks. To evaluate the effects of DPP4 inhibition on inflammation, a subset of mice were fed the HFFC or CD supplemented with the highly selective DPP4 inhibitor, MK-0626 (15.5 mg per 4056 kcal of diet, Merck & Co.)^27^ for 2 or 14 weeks. A time line of the diet regimen is provided in Supplementary Fig. 1a. Body weights were increased in mice fed HFFC vs CD (Supplementary Fig. 1D); however, in keeping with the weight neutral effects of DPP4i treatment, 2 or 14 weeks of MK-0626 had no impact on body weight in either CD or HFFC diet-fed mice (Supplementary Fig. 1D). Eight week-old *Glp1r*^*+/+*^ and *Glp1r*^−/−^ mice were maintained on the HFFC or CD for 11 weeks. After 9 weeks, a subset of the HFFC diet group was switched to HFFC diet containing MK-0626 for an additional 2 weeks. A time line of the diet schedule for *Glp1r*^*+/+*^ and *Glp1r*^−/−^ mice is presented in Supplementary Fig. 8A).

### Preparation of protein extracts from mouse tissues

Tissue samples were homogenised in lysis buffer (50 mM Tris HCl, pH 8.0, 1 mM EDTA, 10% glycerol, 0.02% Brij-35 (Sigma #B4184)) using the TissueLyser II System (Qiagen). Tissues for cytokine assays were homogenised in the above lysis buffer supplemented with 1 mM dithiothreitol and protease inhibitors. Protein lysates were kept on ice for 30 min and then cleared by centrifugation at 5000 × *g* for 10 min. The total protein concentration in each sample was measured by Bradford protein assay.

### Plasma samples

For active GIP and GLP-1 assays, mouse plasma was prepared from tail blood samples collected from mice that had been fasted for 5 h (08:00 a.m.-01:00 p.m.). For all other analyses, mouse plasma was prepared from non-fasting blood samples obtained by cardiac puncture at sacrifice or from tail vein blood samples collected from live mice into heparin-coated capillary microvette tubes (Sarstedt). Blood samples were mixed with a 10% volume of 40 mM EDTA (for measurement of DPP4 activity and protein) or TED (5000 KIU/ml Aprotinin (Sigma #A6279), 1.2 mg/ml EDTA and 0.1 mM Diprotin A (Sigma #I9759) for measurement of active GIP and GLP-1 and cytokine levels). Plasma was separated from blood samples by centrifugation at 18,000 × *g* for 5 min at 4 °C and stored at −80 °C. Human plasma samples from 600 individuals were selected from a subset of patients that participated in the Trial Evaluating Cardiovascular Outcomes with Sitagliptin (TECOS) study^30^ ClinicalTrials.gov Identifier: NCT00790205 and provided consent for exploratory biomarker analysis. Two plasma samples (at screening and 12 months after sitagliptin or placebo initiation) were obtained from each patient in the following treatment groups: (1) 150 patients randomised to placebo and no metformin use at baseline or through 12 months, (2) 150 patients randomised to the DPP4 inhibitor sitagliptin (100 mg/day) and no metformin use at baseline or through 12 months, (3) 150 patients randomised to placebo and taking metformin at baseline and (4) 150 patients randomised to sitagliptin (100 mg/day) and taking metformin at baseline. All patients received standard medical care in addition to the above treatments. Patient samples were chosen by selecting matched pairs for baseline metformin and no metformin, where matches were based on exact matches of randomised treatment and obesity class and nearest match of age, sex, diabetes duration, BMI and baseline HbA1c. All assays on human plasma samples were performed by individuals who were blinded in regard to treatment groups. Patient samples excluded from analyses included four that were haemolysed, two screening samples that were blood, one missing screening sample and one 12-month sample that was blood. Patient baseline characteristics are listed in Supplementary Table 1.

### Pre-defined exploratory endpoints for TECOS plasma samples

Informed consent for exploratory analyses was obtained from participants participating in the TECOS trial, as described in the original TECOS publication^30^ which includes a list of the participating institutions, governed by their respective research ethics boards^30^. The current pre-defined exploratory analysis was approved by the TECOS Publication Steering Committee. The current studies complied with all relevant ethical regulations for work with human participants. The pre-defined primary hypothesis examined whether sitagliptin therapy was associated with induction of sDPP4 and inflammatory biomarkers in the TECOS trial. Secondary exploratory hypotheses tested potential links between metformin use, BMI and plasma levels of DPP4 activity, sDPP4 and inflammatory biomarkers.

### DPP4 activity assay

DPP4 activity levels were measured in 10 μl of mouse or human plasma or 25 μl of mouse tissue protein extract (undiluted for bone marrow and diluted 1/10 to 1/50 for other tissues) via fluorometric assay (AMC standard (Bachem #Q1025), H-Gly-Pro-AMC HBr substrate (Bachem #I-1225). DPP4 activity in tissue was normalised to total protein content.

### DPP4 protein assay

Levels of DPP4 protein were measured in 100 μl of mouse plasma (diluted 1/150) or tissue protein extract (diluted 1/100 to 1/3000) using the Mouse DPPIV/CD26 DuoSet ELISA kit (R&D Systems #DY954). Tissue DPP4 protein levels were normalised to total protein content. DPP4 protein levels were assayed in 100 μl of human plasma (diluted 1/1000) using the Human DPPIV/CD26 DuoSet ELISA kit (R&D Systems #1180). DPP4 protein levels were also measured in a subset of 80 human plasma samples (40 baseline and 40 month 12) using the RayBio® Human CD26 ELISA kit (diluted 1/1000; RayBiotech #ELH-CD26) and the Human sCD26 ELISA kit (diluted 1/5; Thermo Fisher #BMS235). Plasma DPP4 protein levels are referred to as soluble DPP4 (sDPP4) throughout the manuscript.

### RNA isolation, cDNA synthesis and mRNA expression

Total RNA was extracted from tissues using Tri Reagent (Molecular Research Center Inc.) and the TissueLyser II System (Qiagen). cDNA was synthesised from DNase I-treated (Thermo Fisher) total RNA (0.5-2 μg) using random hexamers and Superscript III (Thermo Fisher). Quantification of mRNA levels was carried out using the QuantStudio 5 Real-Time PCR System (Thermo Fisher) and TaqMan Gene Expression Assays (Thermo Fisher). Relative mRNA levels were calculated using the 2^−ΔCt^ method, with cyclophilin (*Ppia*), TATA box protein (*Tbp*), or ribosomal protein L32 (*Rpl32*) used for normalisation. A list of TaqMan Gene Expression Assays and associated ID numbers is provided in Supplementary Table 4.

### Cytokine assays

Cytokine levels were measured in 25 μl of mouse plasma or tissue extract (diluted 1/2) by immunoassay using the V-PLEX Mouse Proinflammatory Panel1 Kit (Mesoscale #K15048D). MSD V-PLEX human assay kits (Mesoscale) were used to measure levels of MCP-1 (Mesoscale #K151NND, 1/4 sample dilution), CRP (Mesoscale # K151STD, 1/1000 sample dilution) and IL-6 and TNFα (Mesoscale #K15049D, 1/2 sample dilution) in human plasma samples. The same control sample was run on all assay plates to monitor assay variability.

### Active GIP and GLP-1 assays

Levels of active GIP and GLP-1 were measured after 5 h of fasting in plasma samples obtained from WT mice maintained on CD or HFFC diet with or without 14 weeks of MK-0626 using the Mouse Active GIP ELISA kit (Crystal Chem #81511) and the V-Plex Active GLP-1 kit (Mesoscale #K1503OD).

### Mouse bone marrow transplant studies

Bone marrow chimeras were generated by lethal total body irradiation (1200 cGy, split into two equal doses 4 h apart) of 8–10 week-old CD45.2^+^ *Dpp4*^EC+/+^ and *Dpp4*^EC−/−^ recipient female mice, followed by reconstitution with tail vein injection of 5 × 10^6^ congenic bone marrow cells isolated from WT male CD45.1^+^ B6.SJL-*Ptprc*^a^ *Pepc*^b^/BoyJ (The Jackson Laboratory stock#002014) donor mice. Reconstitution efficiency was assessed 8 weeks later by flow cytometric analysis (Gallios, Beckman Coulter) of blood using CD45.1-PE-Cy5.5 and CD45.2-APC-Cy7 antibodies and determined to be 88–97%. A sham group, consisting of *Dpp4*^EC+/+^ and *Dpp4*^EC−/−^ female mice not subjected to total body irradiation or bone marrow transplant, was used as controls. At 8 weeks post bone marrow transplant, mice were given normal drinking water or drinking water containing sitagliptin (Januvia® 4 g/l, ≈300–350 mg/kg body weight/day) for 7 days. Tail vein blood samples (100 μl) were collected before and at 7 days after initiation of treatment. Plasma was isolated and used for assay of DPP4 activity and protein as described above. On the eighth day, all mice were switched to regular drinking water for a 10-day drug washout period. Following this, treatment groups were switched, and the sitagliptin study was repeated.

### Studies with sitagliptin ± metformin in WT mice

Fourteen-week-old WT C57BL/6J male mice were randomly assigned to the following 7-day treatments: (1) normal drinking water and HFFC diet (control group), (2) normal drinking water and HFFC diet supplemented with sitagliptin (sitagliptin group), (3) drinking water supplemented with metformin and HFFC diet (metformin group) or (4) drinking water supplemented with metformin and HFFC diet supplemented with sitagliptin (metformin plus sitagliptin group). The D09100301 HFFC diet used above was discontinued and replaced by D09100310 (Research Diets). This diet also contains 40% kcal fat, 20% kcal fructose and 2% cholesterol; however the major source of fat is palm oil instead of Primex shortening. The dose of sitagliptin in the HFFC diet was 4 g/kg of diet (≈300 mg/kg body weight/day). The dose of metformin in the drinking water was 2.5 g/l (≈300 mg/kg body weight/day). Plasma was prepared from tail vein blood samples (100 μl) collected before and at 3 and 7 days after initiation of treatment. Bone marrow samples were extracted from both femurs when mice were sacrificed after 7 days of treatment. DPP4 activity and protein levels were measured in plasma and bone marrow as described above.

### LPS experiments in WT mice

Fourteen-week-old WT C57BL/6J mice were fed either HFFC diet or the same diet supplemented with sitagliptin (described in greater detail above) for four days. On the evening of the third day (approximately 4 pm) mice were given a single ip injection of 35 μg lipopolysaccharide (LPS, from *E. coli* O111:B4, Sigma #L3024) or vehicle (PBS). The following day, mice were given a second ip injection of 35 μg of LPS or vehicle and sacrificed one hour later. DPP4 activity and protein levels were measured in plasma samples prepared from cardiac blood and in bone marrow extracted from femurs as described above.

### Statistics

All mouse data are presented as mean ± SEM. Statistical significance was determined by unpaired two-tailed *t*-test or one-way or two-way ANOVA using GraphPad Prism, version 7.04 (GraphPad). A *P* value ≤ 0.05 was considered to be statistically significant. Human data analyses were performed using SAS® software, version p.4. The associations between sitagliptin and the endpoints of sDPP4, DPP4 activity, IL-6, CRP, MCP-1 and TNFα, measured at 12 months after baseline, were evaluated using linear regression models with sitagliptin as the covariate of interest and adjustment covariates including baseline value of the biomarker of interest, age, sex, BMI, baseline HbA_1c_ and diabetes duration. The endpoints in linear regression models were log2 transformed. Continuous covariates that violated the assumption of normality were included using restricted cubic splines transformations. Least squares means for each treatment group and the difference in least squares means were estimated for each biomarker. Spearman correlation coefficients were used to assess the relationships among biomarkers at 12 months, and where there was evidence of association scatterplots were produced.

### Study approval

All mouse experiments were carried out in accordance with protocols and guidelines approved by the Animal Care Committee at the Toronto Centre for Phenogenomics (approval #20-0045H). The use of human plasma samples from TECOS for the purposes of measuring DPP4 activity, DPP4 protein and cytokine levels was approved by Merck in conjunction with the TECOS Steering Committee.

### Reporting summary

Further information on research design is available in the Nature Research Reporting Summary linked to this article.

## Supplementary information

Supplementary Information

Reporting Summary

## Data Availability

Access to the data from the human TECOS trial is subject to application made via submission to the TECOS Trial Steering Committee. Source data are provided with this paper.

## Source data

Source data
