# Supplementary material for: Plasma levels of DPP4 activity and sDPP4 are dissociated from inflammation in mice and humans
Source: Nat Commun. 2020 Jul 28;11:3766. doi: 10.1038/s41467-020-17556-z (PMC7387453; doi:10.1038/s41467-020-17556-z)
Supplement: Supplementary file 1 — Supplementary Information [file 41467_2020_17556_MOESM1_ESM.pdf]

Supplementary Table 1: Baseline participant characteristics according to randomized treatment and baseline metformin

	Sitagliptin and no metformin (N=146)	Sitagliptin and metformin (N=149)	Placebo and no metformin (N=149)	Placebo and metformin (N=148)
**Age at Randomization (yrs)**	68 (60, 72)	67 (61, 73)	68 (62, 73)	70 (62, 74)
**Female sex**	39 (26.7%)	43 (28.9%)	42 (28.2%)	53 (35.8%)
**BMI (kg/m^2)**				
**≤30**	72 (49.3%)	74 (49.7%)	74 (49.7%)	73 (49.3%)
**>30**	74 (50.7%)	75 (50.3%)	75 (50.3%)	75 (50.7%)
**Qualifying HbA1c (%)**	7.3 (7.0, 7.7)	7.2 (6.9, 7.7)	7.3 (6.8, 7.7)	7.4 (6.9, 7.7)
**Duration of type 2 diabetes (yrs)**	12 (7, 21)	13 (6, 20)	13 (8, 22)	12 (6, 20)
**DPP4 activity (mU/mL)**	3.7 (3.2, 4.3)	3.4 (3.0, 3.9)	3.8 (3.3, 4.3)	3.6 (3.1, 4.1)
**sDPP4 protein (ng/mL)**	569 (479, 700)	525 (445, 631)	582 (514, 697)	556 (450, 693)
**CRP (µg/mL)**	2.5 (1.1, 5.5)	1.8 (0.8, 5.0)	2.9 (1.1, 7.1)	2.4 (0.9, 4.0)
**IL-6 (pg/mL)**	1.2 (0.8, 1.9)	0.9 (0.6, 1.3)	1.3 (0.8, 2.0)	1.0 (0.8, 1.5)
**TNFa (pg/mL)**	2.7 (2.2, 3.3)	2.7 (2.1, 3.2)	2.9 (2.1, 3.5)	2.7 (2.2, 3.3)
**MCP-1 (pg/mL)**	171 (135, 208)	166 (144, 197)	182 (149, 213)	161 (137, 189)

Data are summarized as number (percentage) for categorical variables and median (25th, 75th percentile) for continuous variables. BMI, body mass index.

Supplementary Table 2: **Association of baseline metformin and baseline biomarkers adjusted for clinical covariates**

Baseline log base-2 transformed biomarker	N	Least squares means for no baseline metformin	Least squares means for baseline metformin use	Difference in least squares means	F	p
**sDPP4**	592	9.20 (9.15, 9.25)	9.11 (9.06, 9.16)	-0.09 (-0.16, -0.02)	7.27	0.007
**DPP4 activity**	592	1.88 (1.84, 1.93)	1.80 (1.75, 1.84)	-0.09 (-0.15, -0.02)	6.73	0.010
**CRP**	592	1.51 (1.32, 1.71)	1.12 (0.92, 1.32)	-0.39 (-0.67, -0.11)	7.59	0.006
**IL-6**	592	0.35 (0.23, 0.47)	0.05 (-0.07, 0.17)	-0.30 (-0.48, -0.12)	11.28	<0.001
**TNFa**	592	1.49 (1.43, 1.55)	1.37 (1.31, 1.43)	-0.12 (-0.20, -0.03)	7.02	0.008
**MCP-1**	592	7.46 (7.41, 7.52)	7.37 (7.32, 7.43)	-0.09 (-0.17, -0.01)	5.31	0.022

P values are two-sided and derived from multiple linear regression models. No adjustments were made for multiple comparisons.

Supplementary Table 3. Correlation of sDPP4 protein with other markers at 12 months

Month 12 biomarker	N	Spearman correlation	p-value
**DPP-4 activity (mU/mL)**	592	0.38	<0.001
**CRP (µg/mL)**	592	-0.06	0.158
**IL-6 (pg/mL)**	592	-0.11	0.005
**TNFa (pg/mL)**	592	-0.05	0.261
**MCP-1 (pg/mL)**	592	-0.03	0.430

P values are two-sided and derived from Spearman correlation. No adjustments were made for multiple comparisons.

Supplementary Table 4: qPCR primers for Taqman gene epression

Gene	Description	Assay ID
Adgre1	Adhesion G protein-coupled receptor E1	Mm00802529_m1
Alpi	Alkaline phosphatase, intestinal	Mm01285814_g1
Cav 1	Caveolin 1	Mm00483057_m1
Ccl2	C-C motif chemokine ligand 2	Mm00441242_m1
CD36	Cluster of differentiation 36	Mm00432403_m1
Col1a1	Collagen, type 1, alpha 1	Mm00801666_g1
Cyp7a1	Cytochrome P450 Family 7 Subfamily A Member 1	Mm00484152_m1
Egfr	Epidermal growth factor receptor	Mm00433023_m1
Fgfr4	Fibroblast growth factor receptor 4	Mm01341852_m1
Gip	Glucose-dependent insulinotropic polypeptide	Mm00433601_m1
Havcr1	Hepatitis A Virus Cellular Receptor 1	Mm00506686_m1
Icam1	Intercellular Adhesion Molecule 1	Mm00516023_m1
Ifng	Interferon gamma	Mm01168134_m1
Il10	Interleukin 10	Mm99999062_m1
Il12b	Interleukin 12b	Mm99999067_m1
Il18	Interleukin 18	Mm00434226_m1
Il1b	Interleukin 1b	Mm01336189_m1
Il25	Interleukin 25	Mm00499822_m1
Il33	Interleukin 33	Mm00505403_m1
Il6	Interleukin 6	Mm00446190_m1
Lcn2	Lipocalin 2	Mm01324470_m1
Lipc	Lipase C, hepatic type	Mm01171487_m1
Ocln	Occludin	Mm00500912_m1
Ppia	Peptidylprolyl isomerase A	Mm02342430_g1
Reg3a	Regenerating family member 3 alpha	Mm00441127_m1
Reg3b	Regenerating family member 3 beta	Mm00440616_g1
Reg3g	Regenerating family member 3 gamma	Mm00441127_m1
Rpl32	Ribosomal protein L32	Mm02528467_g1
Tbp	Tata box binding protein	Mm00446973_m1
Tff2	Trefoil factor 2	Mm00447491_m1
Tff3	Trefoil factor 3	Mm00495590_m1
Tgfbb1	Transforming growth factor beta 1	Mm01178820_m1
Tjp1 (ZO-1)	Tight junction protein 1	Mm00493699_m1
Tlr4	Toll-like receptor 4	Mm00445273_m1
Tnfa	Tumour necrosis factor alpha	Mm00443258_m1
Tslp	Thymic stromal lymphopoietin	Mm00498739_m1

All primers are from Thermo-Fisher Scientific.

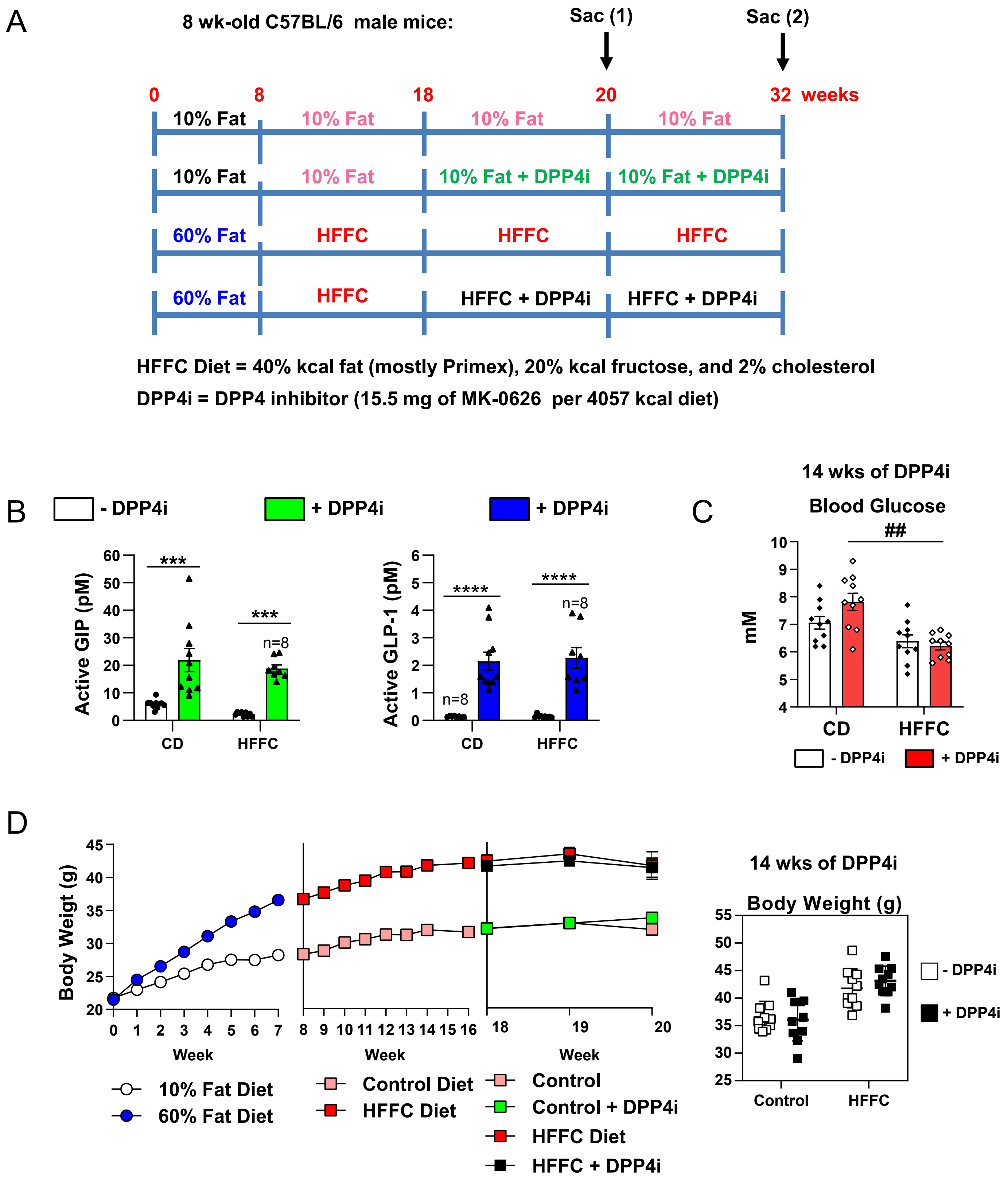

Supplementary Figure 1

Supplementary Figure 1. Long-term inhibition of DPP4 increases plasma levels of active GIP and GLP-1 but does not modify HFFC diet-induced weight gain in WT mice. (A) Schematic depicting the diet regimen and time of sacrifice (Sac) in the WT mouse studies. (B) Five hour fasted plasma levels of active gastric inhibitory polypeptide (GIP) and glucagon-like peptide -1 (GLP-1) in WT mice that were maintained on control diet (CD) or high fat, fructose and cholesterol (HFFC) diet supplemented with (+) or without (-) the DPP4 inhibitor (DPP4i) MK-0626 for 14 wks. (C) Non-fasting blood glucose levels at the time of sacrifice in WT mice that were maintained on CD or HFFC diet supplemented with or without DPP4i. (D) Weekly body weights and body weights at the time of sacrifice in WT mice that were maintained on CD or HFFC diet supplemented with or without DPP4i. Data shown are mean $\pm$ SEM. n=9 or 10 mice per group, except where n=8 as indicated on the graph above the data set.****P*<0.001,*****P*<0.0001 vs. DPP4i-treated. ##*P*<0.01 for CD vs. HFFC. Data in (B-D) were analyzed by two-way ANOVA. Source data are provided as a Source data file.

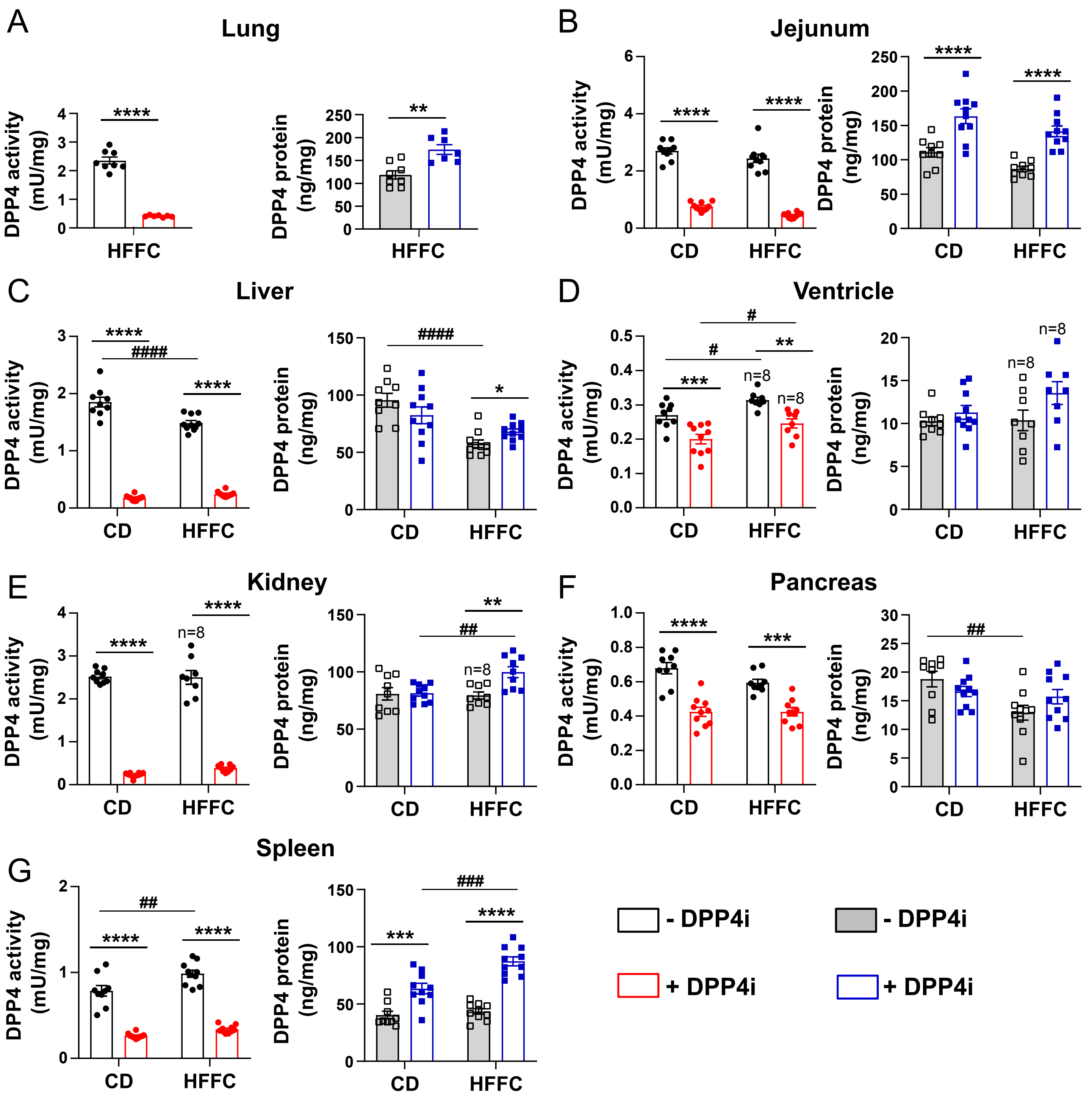

Supplementary Figure 2. DPP4 activity and protein levels in tissues after 2 or 4 wks of DPP4i. Levels of DPP4 activity (left panels) and protein (right panels) in (A) lung, (B) jejunum, (C) liver, (D) whole ventricle, (E) kidney, (F) pancreas and (G) spleen from mice that were maintained on control diet (CD) or a high fat, fructose and cholesterol (HFFC) diet for 10 weeks and then the same diets supplemented with (+) or without (-) the DPP4 inhibitor (DPP4i) MK-0626 for 4 (A) or 2 (B-G) wks. Data shown are mean ± SEM. For A, n=7 or 8 mice per group. For B-G, n=9 or 10 mice per group, except where n=8 as indicated on the graph above the data set. *P<0.05, **P<0.01, ***P<0.001, ****P<0.0001 vs. DPP4i-treated. #P<0.05, ##P<0.01, ###P<0.001, ####P<0.0001 for CD vs. HFFC. Data were analyzed by two-sided unpaired Student's t-test (A) or two way ANOVA (B-G). Source data are provided as a Source data file.

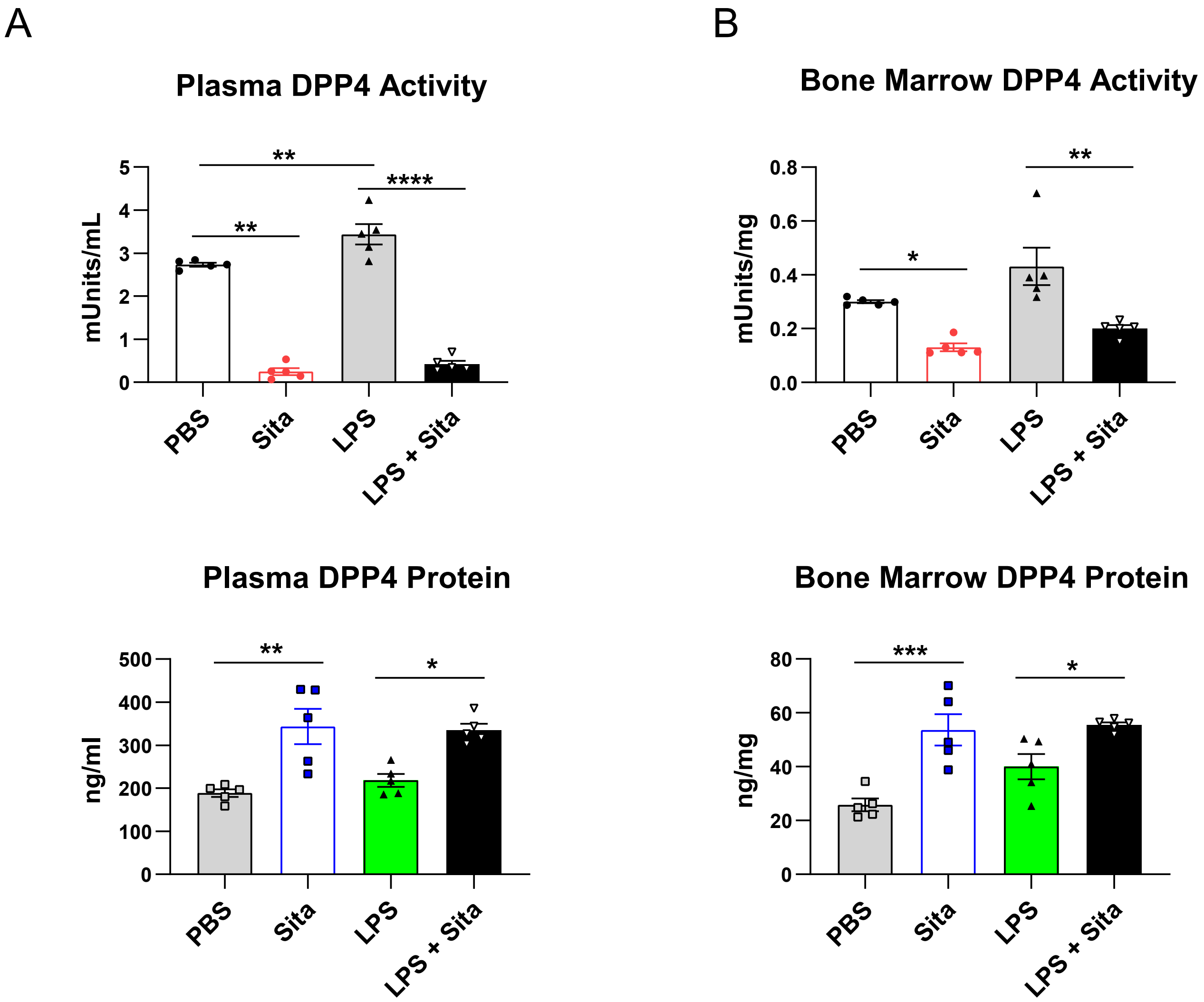

Supplementary Figure 3. Sitagliptin reduces DPP4 activity and increases DPP4 protein levels in plasma and bone marrow of LPS-treated mice. Levels of DPP4 activity (top panel) and protein (bottom panel) in (A) plasma and (B) bone marrow from WT mice that were fed a HFFC diet for 4 days and treated with LPS (two injections of 35 μg each, one on the evening prior to sacrifice and the second one hour before sacrifice) or PBS vehicle. n=5 mice per group. Data shown are mean ± SEM. **P*<0.05, ***P*<0.01, ****P*<0.001, *****P*<0.0001 vs. the indicated comparator. Data were analyzed by two-way ANOVA. Source data are provided as a Source data file.

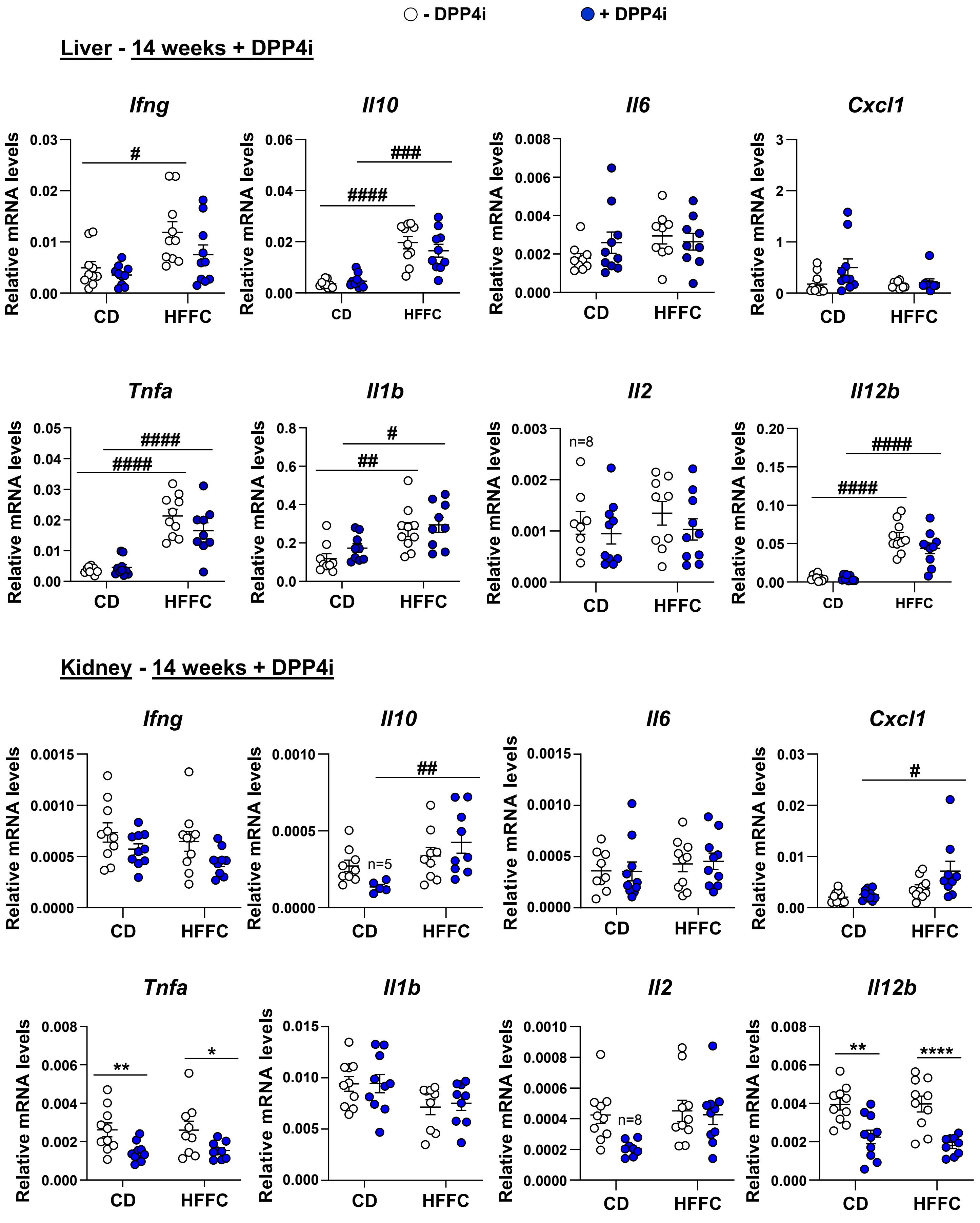

Supplementary Figure 4

Supplementary Figure 4. Gene expression in mouse liver and kidney after 14 wks of DPP4i.

mRNA levels of genes central to inflammation were examined in liver (upper panels) and kidney (lower panels) samples from WT mice that were maintained on control diet (CD) or a high fat, fructose and cholesterol (HFFC) diet for 10 weeks and then the same diets supplemented with (+) or without (-) the DPP4 inhibitor (DPP4i) MK-0626 for 14 wks. Data were normalized to *Tbp* (liver) or *Rpl32* (kidney) expression and are mean $\pm$ SEM. n=9 or 10 mice per group, except where the specific n is indicated on the graph above the data set. **P*<0.05, ***P*<0.01, *****P*<0.0001 vs. DPP4i-treated. #*P*<0.05, ##*P*<0.01, ###*P*<0.001, ####*P*<0.0001 for CD vs. HFFC. Data were analyzed by two-way ANOVA. Source data are provided as a Source data file.

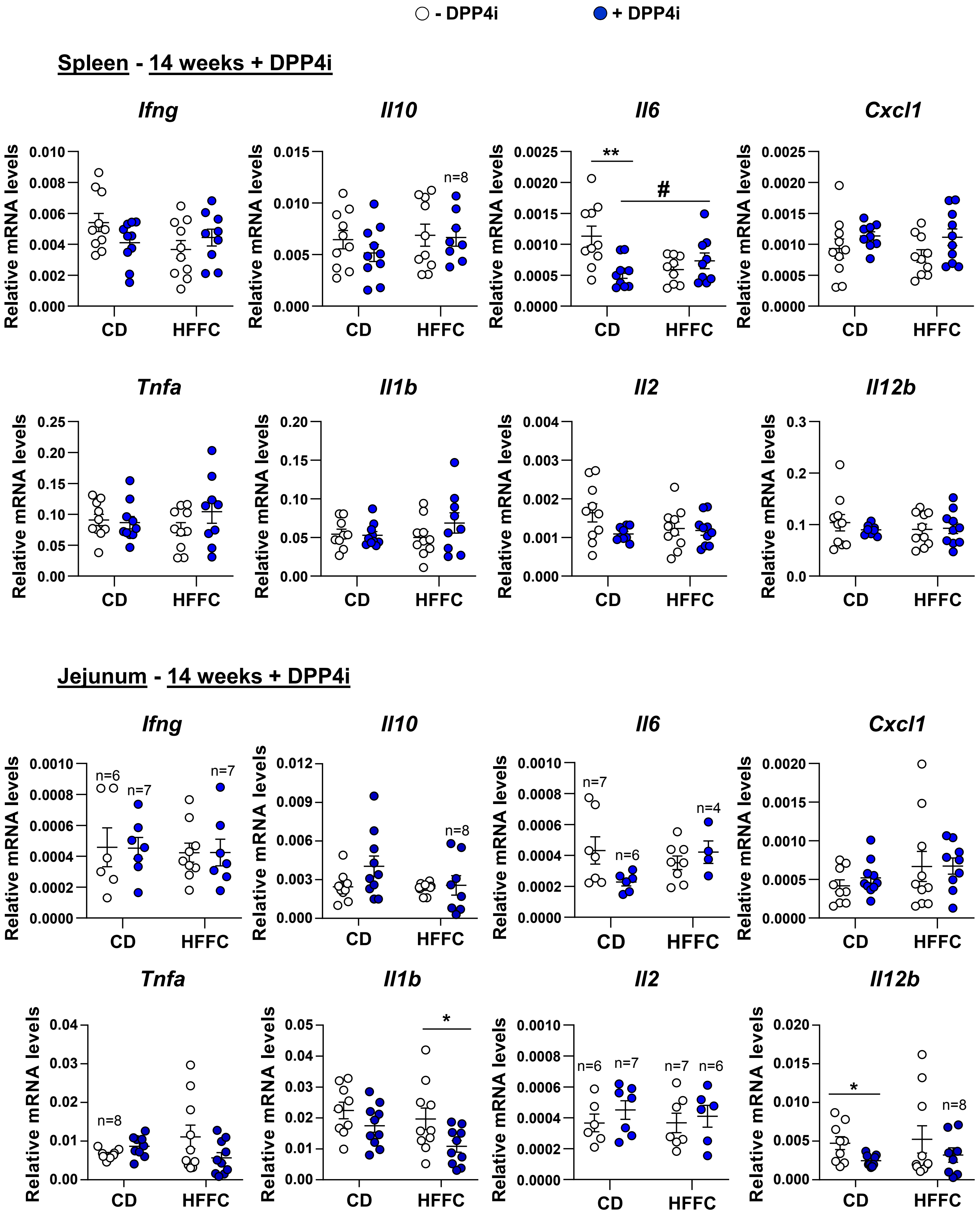

Supplementary Figure 5

Supplementary Figure 5. Gene expression in mouse spleen and jejunum after 14 wks of DPP4i.
mRNA levels of genes central to inflammation were examined in spleen (upper panels) and jejunum (lower panels) samples from WT mice that were maintained on control diet (CD) or a high fat, fructose and cholesterol (HFFC) diet for 10 weeks and then the same diets supplemented with (+) or without (-) the DPP4 inhibitor (DPP4i) MK-0626 for 14 wks. Data were normalized to *Rpl32* expression and are mean $\pm$ SEM. n=9 or 10 mice per group, except where the specific n is indicated on the graph above the data set. **P*<0.05, ***P*<0.01 vs. DPP4i-treated. #*P*<0.05 for CD vs. HFFC. Data were analyzed by two-way ANOVA. Source data are provided as a Source data file.

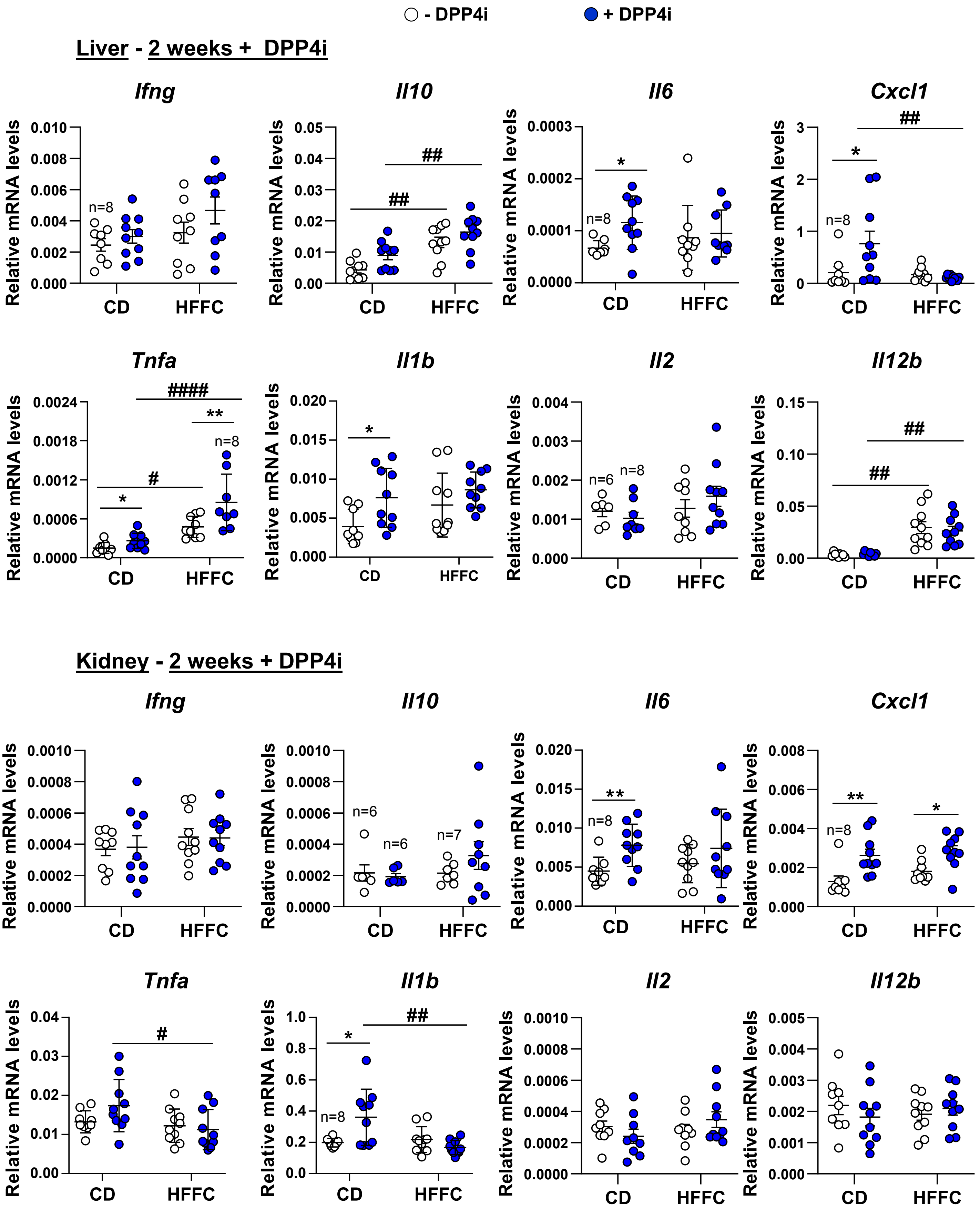

Supplementary Figure 6

Supplementary Figure 6. Gene expression in mouse liver and kidney after 2 wks of DPP4i.
mRNA levels corresponding to genes important for inflammation were examined in liver (upper panels and kidney (lower panels) samples from WT mice that were maintained on control diet (CD) or a high fat, fructose and cholesterol (HFFC) diet for 10 weeks and then the same diets supplemented with (+) or without (-) the DPP4 inhibitor (DPP4i) MK-0626 for 2 wks. Data were normalized to *Tbp* (liver) or *Rpl32* (kidney) expression and are mean $\pm$ SEM. n=9 or 10 mice per group, except where the specific n is indicated on the graph above the data set. *P<0.05, **P<0.01 vs. DPP4i-treated. #P<0.05, ##P<0.01, ####P<0.0001 for CD vs. HFFC. Data were analyzed by two-way ANOVA. Source data are provided as a Source data file.

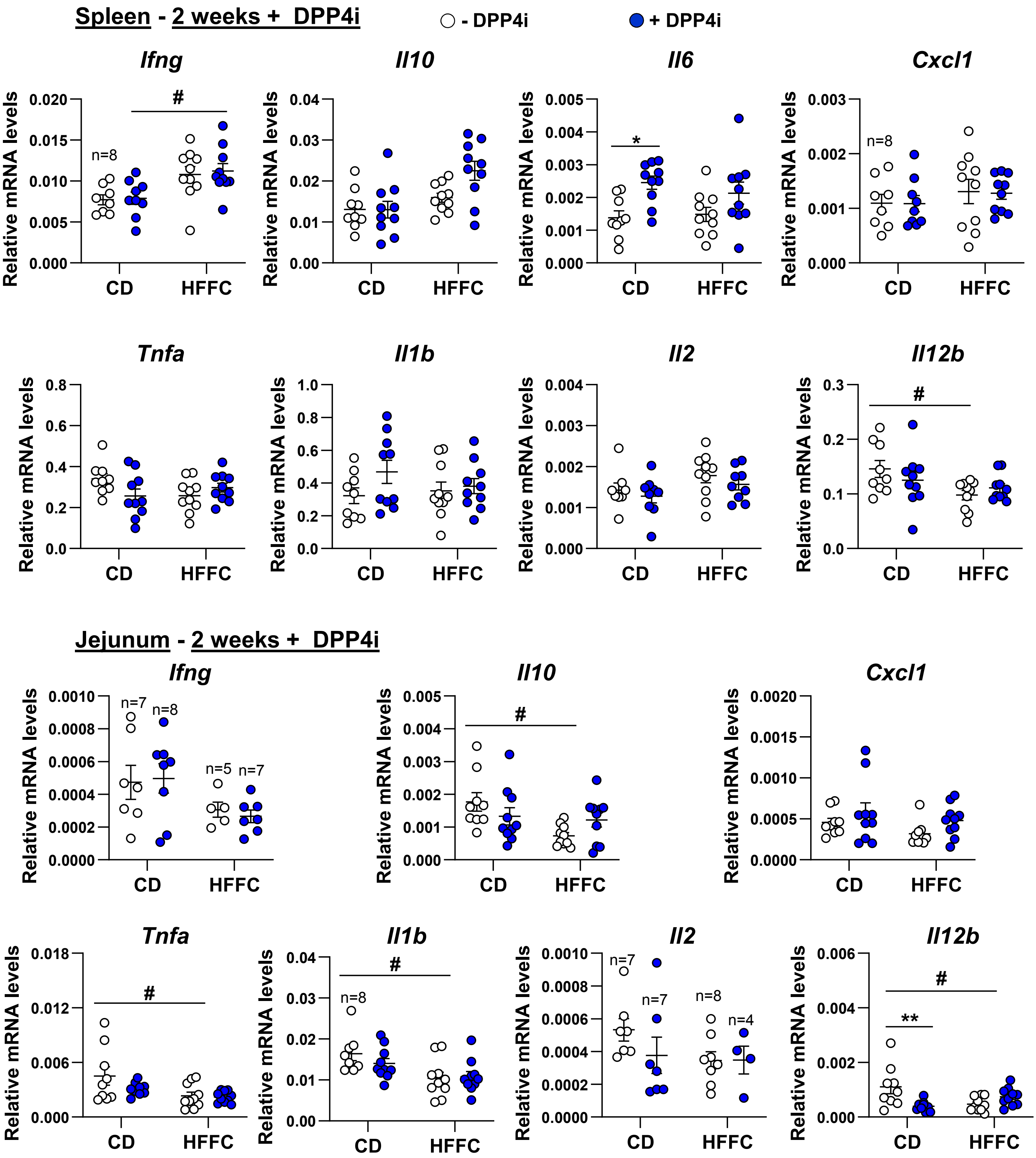

Supplementary Figure 7. Gene expression in mouse spleen and jejunum after 2 wks of DPP4i. mRNA levels of genes central to inflammation were examined in spleen (upper panels) and jejunum (lower panels) samples from WT mice that were maintained on control diet (CD) or a high fat, fructose and cholesterol (HFFC) diet for 10 weeks and then the same diets supplemented with (+) or without (-) the DPP4 inhibitor (DPP4i) MK-0626 for 2 wks. Data were normalized to *Rpl32* expression and are mean ± SEM. n=9 or 10 mice per group, except where the specific n is indicated on the graph above the data set. **P*<0.05, ***P*<0.01 vs. DPP4i-treated. #*P*<0.05 for CD vs. HFFC. Data were analyzed by two-way ANOVA. Source data are provided as a Source data file.

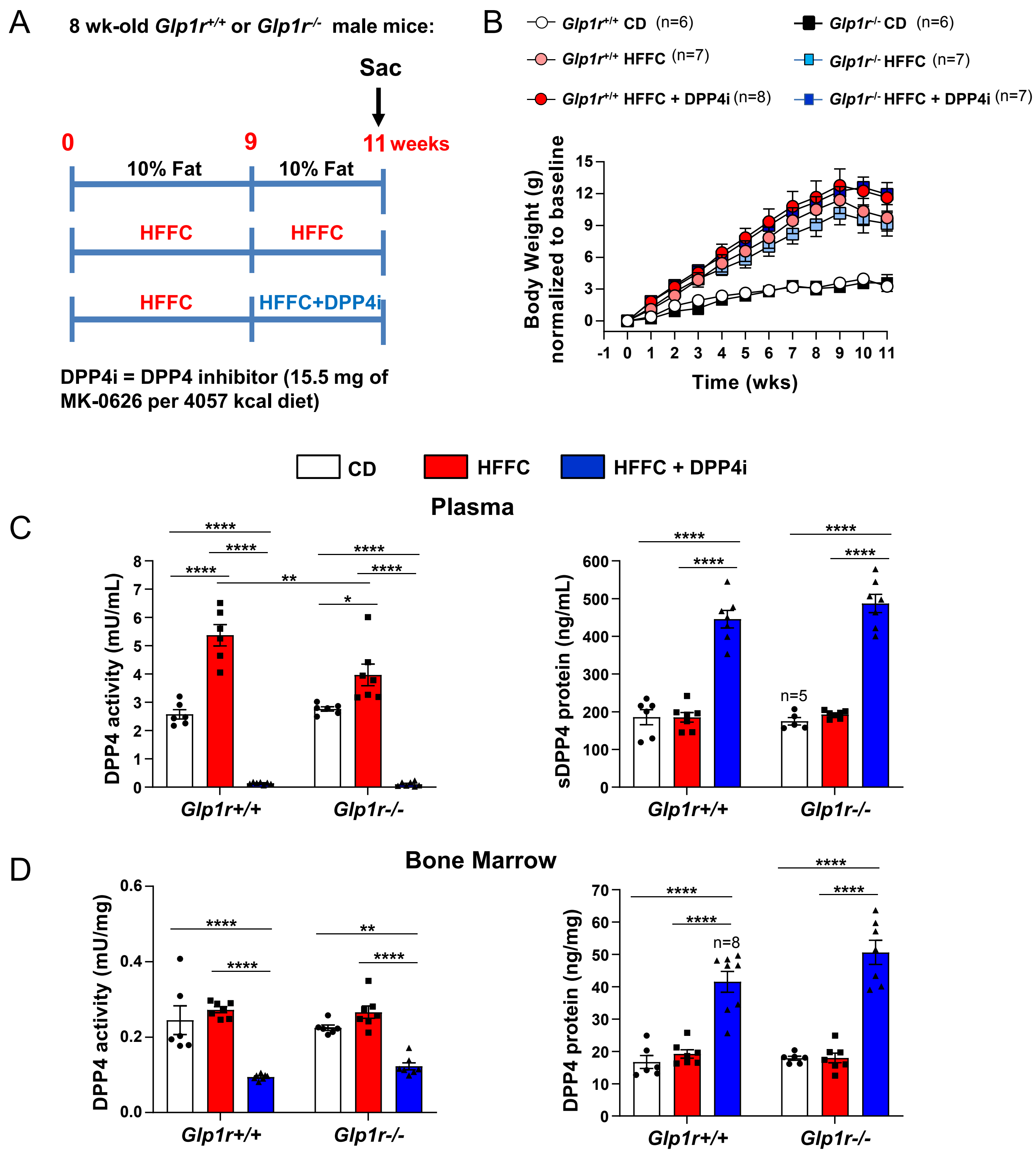

Supplementary Figure 8. (A) Schematic depicting the diet regimen and time of sacrifice (Sac) in the *Glp1r*$^{+/+}$ and *Glp1r*$^{-/-}$ mouse studies. (B) Weekly body weights of *Glp1r*$^{+/+}$ and *Glp1r*$^{-/-}$ mice maintained on a control diet (CD) or a high fat, fructose and cholesterol (HFFC) diet for 11 wks, or HFFC diet for 9 wks and then switched to HFFC diet supplemented with (+) the DPP4 inhibitor (DPP4i) MK-0626 (MK) for 2 wks. (C-D) Levels of DPP4 activity (left panels) and protein (right panels) in (C) plasma and (D) bone marrow from *Glp1r*$^{+/+}$ and *Glp1r*$^{-/-}$ mice maintained on CD or HFFC diet for 11 wks +/- DPP4i for the final 2 wks. Data shown are mean ± SEM. For C and D, n=6 or 7, except where the specific n is indicated on the graph above the data set. **P*<0.05, ***P*<0.01, *****P*<0.0001 vs. the indicated comparator. Data were analyzed by two-way ANOVA. Source data are provided as a Source data file.

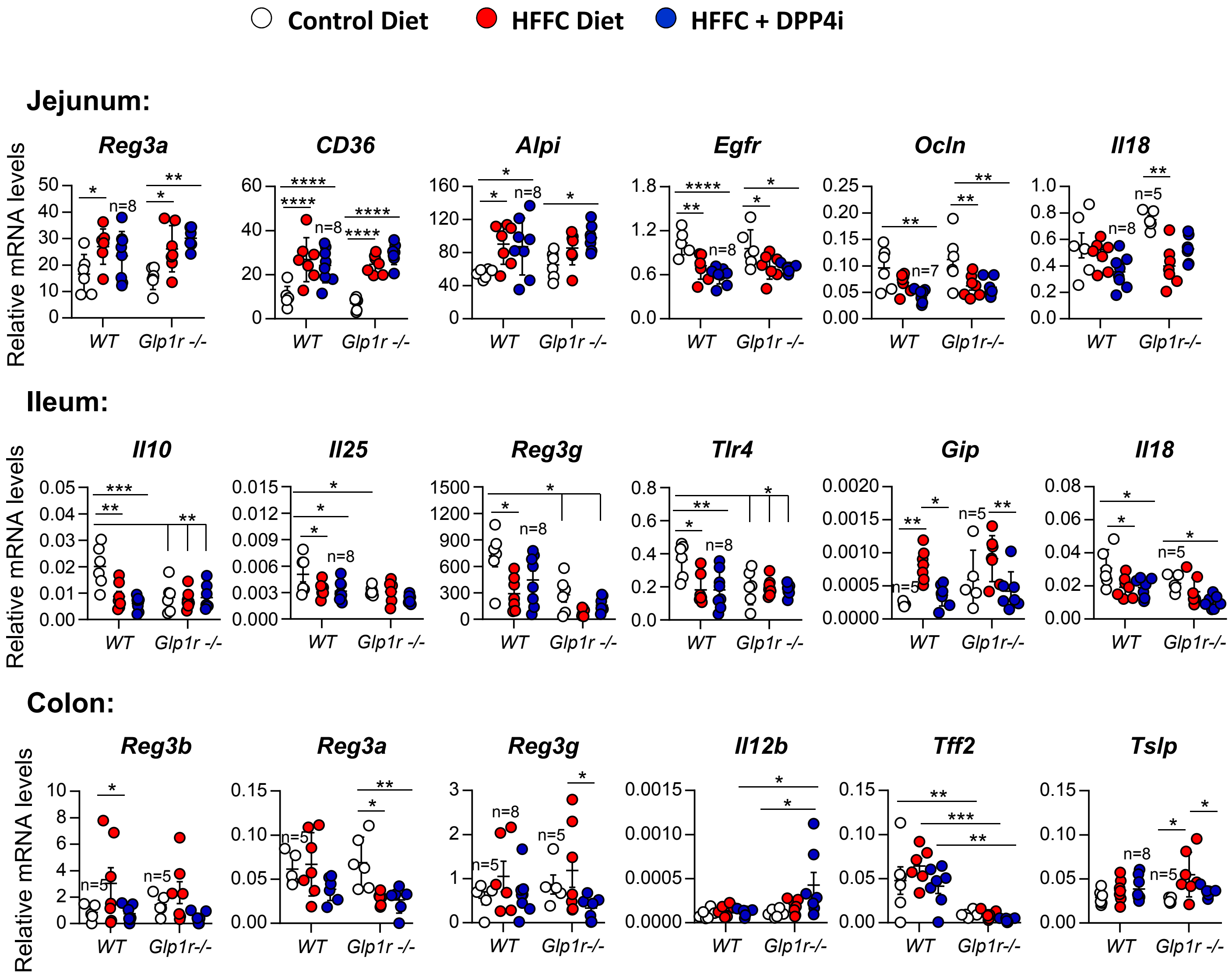

Supplementary Figure 9. Gene expression in *Glp1r*+/+ and *Glp1r*-/- mouse intestine. mRNA levels of genes important for inflammation or epithelial protection and repair were assessed in jejunum, ileum and colon samples from *Glp1r*+/+ (WT) and *Glp1r*-/- mice maintained on a control diet (10% fat) or a high fat, fructose and cholesterol (HFFC) diet for 11 wks, or HFFC diet for 9 wks and then switched to HFFC diet supplemented with (+) the DPP4 inhibitor (DPP4i) MK-0626 for 2 wks. Data were normalized to *Ppia* or *Tbp* and are mean ± SEM. n=6 or 7 mice per group, except where the specific n is indicated on the graph above the data set. **P*<0.05, ***P*<0.01, ****P*<0.001, *****P*<0.0001 vs. the indicated comparator. Data were analyzed by two-way ANOVA. Source data are provided as a Source data file.

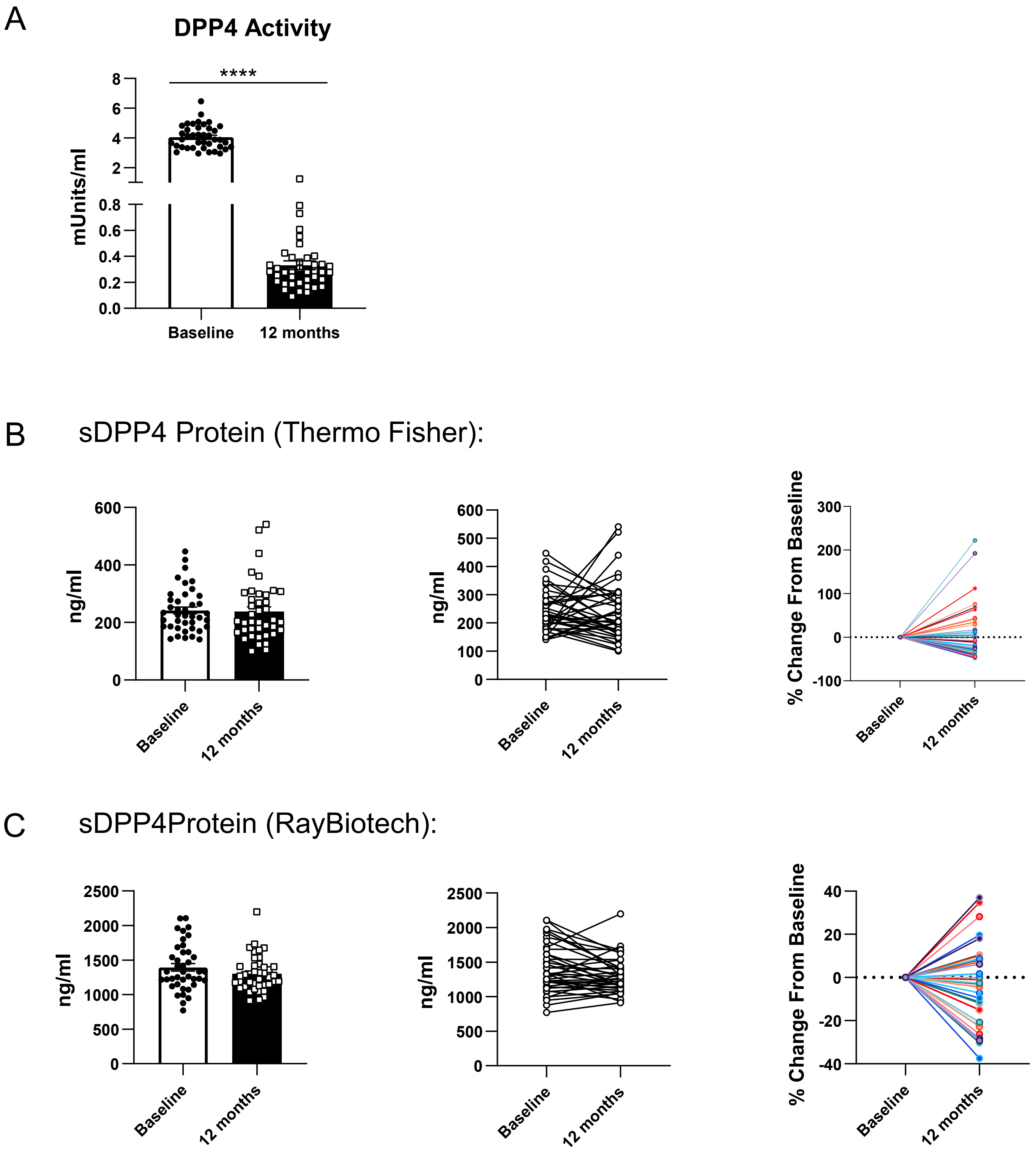

Supplementary Figure 10. Plasma levels of sDPP4 vary widely among TECOS trial patients.
(A) DPP4 activity and (B, C) sDPP4 protein levels at baseline and month 12 in plasma samples from a subset of patients from the TECOS trial (n=40 patients) that received sitagliptin. Plasma sDPP4 levels were measured using assay kits from two independent sources (B, C). In B and C, data are shown as individual values in a scatter plot (left panels), summary data with a line connecting the baseline data to the 12 month data (center panels), or percent change from baseline (right panels). Data are mean ± SEM. ****P<0.0001 vs. the indicated comparator. Data were analyzed by two-sided unpaired Student's t-test.
