# Supplementary material for: Plasma levels of DPP4 activity and sDPP4 are dissociated from inflammation in mice and humans
Source: Nat Commun. 2020 Jul 28;11:3766. doi: 10.1038/s41467-020-17556-z (PMC7387453; doi:10.1038/s41467-020-17556-z)
Supplement: Supplementary file 2 — Reporting Summary [file 41467_2020_17556_MOESM2_ESM.pdf]

nature research

Corresponding author(s): Laurie L. Baggio, Elodie M. Varin, Jacqueline A. Koehler, Xiemin Cao, Yuliya Lokhnygina, Susanna R. Stevens, Rury R. Holman, and Daniel J. Drucker.

Last updated by author(s): Oct 28, 2019

Reporting Summary

Nature Research wishes to improve the reproducibility of the work that we publish. This form provides structure for consistency and transparency in reporting. For further information on Nature Research policies, seeAuthors & Referees and theEditorial Policy Checklist .

Statistics

For all statistical analyses, confirm that the following items are present in the figure legend, table legend, main text, or Methods section.

n/a	Confirmed	
☐	☒	The exact sample size (*n*) for each experimental group/condition, given as a discrete number and unit of measurement
☐	☒	A statement on whether measurements were taken from distinct samples or whether the same sample was measured repeatedly
☐	☒	The statistical test(s) used AND whether they are one- or two-sided<br>*Only common tests should be described solely by name; describe more complex techniques in the Methods section.*
☐	☒	A description of all covariates tested
☐	☒	A description of any assumptions or corrections, such as tests of normality and adjustment for multiple comparisons
☐	☒	A full description of the statistical parameters including central tendency (e.g. means) or other basic estimates (e.g. regression coefficient) AND variation (e.g. standard deviation) or associated estimates of uncertainty (e.g. confidence intervals)
☐	☒	For null hypothesis testing, the test statistic (e.g. *F*, *t*, *r*) with confidence intervals, effect sizes, degrees of freedom and *P* value noted<br>*Give P values as exact values whenever suitable.*
☒	☐	For Bayesian analysis, information on the choice of priors and Markov chain Monte Carlo settings
☒	☐	For hierarchical and complex designs, identification of the appropriate level for tests and full reporting of outcomes
☒	☐	Estimates of effect sizes (e.g. Cohen's *d*, Pearson's *r*), indicating how they were calculated

Our web collection on statistics for biologists contains articles on many of the points above.

Software and code

Policy information about availability of computer code

Data collection	No software was used.
Data analysis	GraphPad Prism version 7.04 was used for statistical analysis of all mouse data. SAS software version p.4 was used for statistical analysis of all human data.

For manuscripts utilizing custom algorithms or software that are central to the research but not yet described in published literature, software must be made available to editors/reviewers. We strongly encourage code deposition in a community repository (e.g. GitHub). See the Nature Research guidelines for submitting code & software for further information.

Data

Policy information about availability of data

All manuscripts must include a data availability statement. This statement should provide the following information, where applicable:

- Accession codes, unique identifiers, or web links for publicly available datasets
- A list of figures that have associated raw data
- A description of any restrictions on data availability

Access to the data from the human TECOS trial is subject to application made via submission to the TECOS Trial Steering Committee. All original mouse data from this article is provided in a supplementary Source Data Excel file.

Field-specific reporting

Please select the one below that is the best fit for your research. If you are not sure, read the appropriate sections before making your selection.

Life sciences study design

All studies must disclose on these points even when the disclosure is negative.

Sample size	No sample size calculation was performed.<br>The sample size for human samples was based on availability of plasma samples from patients enrolled in the Trial Evaluating Cardiovascular Outcomes with Sitagliptin (TECOS) study who had given consent for exploratory biomarker analysis.<br>For mouse studies, a minimum of 5 mice per group was used. This was based on historical observations made in our laboratory over the last 20 years conducting similar studies where we find a minimum of 5 animals is required to detect real differences between treatment groups. The sample sizes used in the current study were deemed sufficient due to the ability to detect statistical significance in multiple analyses. In some instances, sample size was limited by mouse availability.
Data exclusions	For studies with human plasma samples, patient samples excluded from analyses included four that were haemolysed, two screening samples that were blood, one missing screening sample, and one 12 month sample that was blood.<br>For mouse studies, significant outliers were identified by Grubb's test and removed from the data set.
Replication	For human assays, the same control sample (which consisted of a pooled sample of patient plasma) was run on all assay plates to monitor assay variability. To control for potential differences in sDPP4 assays, a subset of human samples was analyzed with 3 different sDPP4 ELISA kits. Although absolute values differed among the different kits, the overall results were similar. All attempts at replication were successful.<br>In our mouse studies, DPP4 activity and protein levels in response to DPP4 inhibitor treatment were performed in commercial wild-type mice and Glp-1 receptor knockout mice and their litter mate controls, with very similar results. The experiments performed using wild-type mice were performed once using a large cohort (n=8-10 mice per group). The data from Glp-1 receptor knockout and litter mate controls are the combined results from experiments performed in 3 independent cohorts of mice, each yielding similar results. In addition, plasma DPP4 activity and protein levels were measured following treatment with two different DPP4 inhibitors (MK-0626 and sitagliptin). Treatment with either inhibitor reduced DPP4 activity and increased sDPP4 protein levels in mice. Bone marrow transplant experiments were performed once. LPS experiments were performed once. Experiments with sitagliptin +/- metformin were performed in two independent experiments with similar outcomes.
Randomization	Human plasma samples for analysis were selected by matching pairs for baseline metformin and no metformin, where matches were based on exact matches of randomized treatment and obesity class and nearest match of age, sex, diabetes duration, BMI and baseline HbA1c.<br>Mice were allocated to experimental groups by random allocation of home cages to specific treatments.
Blinding	Investigators were blinded to group allocation during data collection and analysis for both human and mouse studies.

Reporting for specific materials, systems and methods

We require information from authors about some types of materials, experimental systems and methods used in many studies. Here, indicate whether each material, system or method listed is relevant to your study. If you are not sure if a list item applies to your research, read the appropriate section before selecting a response.

Materials & experimental systems

n/a	Involved in the study	
☒	☐	Antibodies
☒	☐	Eukaryotic cell lines
☒	☐	Palaeontology
☐	☒	Animals and other organisms
☐	☒	Human research participants
☐	☒	Clinical data

Methods

n/a	Involved in the study	
☒	☐	ChIP-seq
☒	☐	Flow cytometry
☒	☐	MRI-based neuroimaging

Animals and other organisms

Policy information about studies involving animals; ARRIVE guidelines recommended for reporting animal research

Laboratory animals	All mice used in these studies were Mus musculus on a C57BL/6J genetic background. For all studies, except bone marrow transplant studies, experiments were initiated in male mice that were 6-14 weeks old. For bone marrow transplant experiments, recipient mice were 8-10 week old females and donor mice were 8-12 week old males. The following mouse lines were used in our studies: C57BL/6J (purchased from Jackson Labs), Glp1r-/- and Glp1r+/+ litter mates, Dpp4EC-/- and Dpp4EC+/+ litter mates, and B6.SJL-Ptprca Pepcb/BoyJ (purchased from Jackson Labs).
Wild animals	This study did not involve wild animals.
Field-collected samples	This study did not involve samples collected from the field.

Ethics oversight	All mouse experiments were carried out in accordance with protocols and guidelines approved by the Animal Care Committee at the Toronto Centre for Phenogenomics (approval #20-0045H).

Note that full information on the approval of the study protocol must also be provided in the manuscript.

Human research participants

Policy information about studies involving human research participants

Population characteristics	The baseline characteristics of individuals enrolled in the TECOS trial are described in Bethela, A., et al Regional, age and sex differences in baseline characteristics of patients enrolled in the Trial Evaluating Cardiovascular Outcomes with Sitagliptin (TECOS) Diabetes Obes Metab. 2015 Apr;17(4):395-402.
Recruitment	Recruitment and eligibility have been described in Bethela, A., et al Regional, age and sex differences in baseline characteristics of patients enrolled in the Trial Evaluating Cardiovascular Outcomes with Sitagliptin (TECOS) Diabetes Obes Metab. 2015 Apr;17 (4):395-402.
Ethics oversight	The use of human plasma samples from TECOS for tour exploratory analyses with the purposes of measuring DPP4 activity, DPP4 protein and cytokine levels was approved by Merck in conjunction with the TECOS Steering Committee and consistent with the informed consent form signed by a subset of TECOS participants governing exploratory analyses

Note that full information on the approval of the study protocol must also be provided in the manuscript.

Clinical data

Policy information about clinical studies

All manuscripts should comply with the ICMJEguidelines for publication of clinical research and a completedCONSORT checklist must be included with all submissions.

Clinical trial registration	ClinicalTrials.gov Identifier: NCT00790205
Study protocol	Described in Bethela, A., et al Regional, age and sex differences in baseline characteristics of patients enrolled in the Trial Evaluating Cardiovascular Outcomes with Sitagliptin (TECOS) Diabetes Obes Metab. 2015 Apr;17(4):395-402.
Data collection	Plasma samples for exploratory biomarkers collected after informed consent in the TECOS trial. Patients from 38 countries in 5 regions (North America, Eastern and Western Europe, Asia Pacific and Latin America) participated in the TECOS trial. Data were collected from patients using a combination of study visits and visits to the patient's usual care provider. Patients were enrolled between December 2008 and July 2012. The study was closed in March 2015 with a median follow up of 3.0 years.
Outcomes	In our grant submission to the TECOS trial, we pre-specified outcomes The pre-defined primary hypothesis examined whether sitagliptin therapy was associated with induction of sDPP4 and inflammatory biomarkers in the TECOS trial. Secondary exploratory hypotheses tested potential links between metformin use, BMI, and plasma levels of DPP4 activity, sDPP4, and inflammatory biomarkers.
